# Formation of Mobile Chromatin-Associated Nuclear Foci Containing HIV-1 Vpr and VPRBP Is Critical for the Induction of G2 Cell Cycle Arrest

**DOI:** 10.1371/journal.ppat.1001080

**Published:** 2010-09-02

**Authors:** Jean-Philippe Belzile, Levon G. Abrahamyan, Francine C. A. Gérard, Nicole Rougeau, Éric A. Cohen

**Affiliations:** 1 Laboratory of Human Retrovirology, Institut de Recherches Cliniques de Montréal (IRCM), Montreal, Quebec, Canada; 2 Department of Microbiology and Immunology, Université de Montréal, Montreal, Quebec, Canada; Fred Hutchinson Cancer Research Center, United States of America

## Abstract

HIV-1 Viral protein R (Vpr) induces a cell cycle arrest at the G2/M phase by activating the ATR DNA damage/stress checkpoint. Recently, we and several other groups showed that Vpr performs this activity by recruiting the DDB1-CUL4A (VPRBP) E3 ubiquitin ligase. While recruitment of this E3 ubiquitin ligase complex has been shown to be required for G2 arrest, the subcellular compartment where this complex forms and functionally acts is unknown. Herein, using immunofluorescence and confocal microscopy, we show that Vpr forms nuclear foci in several cell types including HeLa cells and primary CD4+ T-lymphocytes. These nuclear foci contain VPRBP and partially overlap with DNA repair foci components such as γ-H2AX, 53BP1 and RPA32. While treatment with the non-specific ATR inhibitor caffeine or depletion of VPRBP by siRNA did not inhibit formation of Vpr nuclear foci, mutations in the C-terminal domain of Vpr and cytoplasmic sequestration of Vpr by overexpression of Gag-Pol resulted in impaired formation of these nuclear structures and defective G2 arrest. Consistently, we observed that G2 arrest-competent sooty mangabey Vpr could form these foci but not its G2 arrest-defective paralog Vpx, suggesting that formation of Vpr nuclear foci represents a critical early event in the induction of G2 arrest. Indeed, we found that Vpr could associate to chromatin via its C-terminal domain and that it could form a complex with VPRBP on chromatin. Finally, analysis of Vpr nuclear foci by time-lapse microscopy showed that they were highly mobile and stable structures. Overall, our results suggest that Vpr recruits the DDB1-CUL4A (VPRBP) E3 ligase to these nuclear foci and uses these mobile structures to target a chromatin-bound cellular substrate for ubiquitination in order to induce DNA damage/replication stress, ultimately leading to ATR activation and G2 cell cycle arrest.

## Author Summary

HIV-1, the causative agent of AIDS, encodes several proteins termed accessory, which play a critical role in viral pathogenesis. One of these accessory proteins, viral protein R (Vpr), has been found to block normal cell division. This impairment of cell division by Vpr is thought to increase viral replication and to trigger immune cell death. However, how Vpr is able to block cell growth remains unknown. We and other investigators recently showed that Vpr was performing this activity by interacting with a cellular protein complex involved in ubiquitination. Ubiquitination is characterized by the conjugation of a small protein called ubiquitin to various other proteins to regulate their degradation or activities. In this report, we demonstrate that Vpr forms mobile punctuate structures called foci on the DNA of host cells. We also show that formation of these foci by Vpr is required to block cell division. We propose that Vpr recruits the ubiquitination complex to these nuclear foci and uses these mobile structures to target a DNA-bound cellular protein for degradation, resulting in the activation of a host cell response leading to a cell division block. Identification of the unknown cellular factor targeted by Vpr will contribute to the understanding of the role of Vpr during HIV infection and AIDS pathogenesis.

## Introduction

HIV-1 encodes several proteins termed accessory that have been implicated in the modulation of host cell environment to promote efficient viral replication and evasion from innate and acquired immunity [1]. One of these accessory proteins, viral protein R (Vpr), is a small amphipathic protein of 96 amino acids. In addition to being expressed in infected cells, Vpr is packaged into virions through an interaction with the p6 domain of the Gag polyprotein precursor [2], [3], [4]. The molecular structure of Vpr was recently resolved and found to consist of a hydrophobic core comprising three interacting alpha helices flanked by N- and C-terminal flexible domains [5]. Of note, the third alpha helix includes a leucine-rich region essential for the stability of the core and the flexible C-terminus comprises a functionally important stretch of positively charged arginine residues [6]. Several biological functions have been attributed to Vpr including transactivation of the viral long terminal repeat (LTR), enhancement of infection in macrophages, induction of apoptosis, and promotion of a cell cycle arrest at the G2/M phase [7].

Vpr-mediated G2 arrest likely plays an important role *in vivo* for viral replication or pathogenesis given that this activity is highly conserved among primate lentiviruses [8], [9] and since abnormal accumulation of cells in G2/M can be observed in HIV-infected individuals [10]. Indeed, recent studies reported that Vpr upregulated the expression of ligands for the activating NKG2D receptor and promoted natural killer (NK) cell-mediated killing by a process that relied on Vpr ability to induce a G2 arrest, thus suggesting an immunomodulatory role for Vpr that may not only contribute to HIV-1-induced CD4+ T-lymphocyte depletion but may also take part in HIV-1-induced NK cell dysfunction [11], [12]. Several investigators have reported that Vpr-induced cell cycle arrest involves the activation of the ATR (ataxia telangiectasia-mutated and Rad3-related; NM_001184)-mediated G2/M checkpoint [10], [13], [14]. ATR is a kinase of the phosphatidylinositol 3 kinase-like family and is involved in the activation of the G2/M checkpoint and in the coordination of DNA repair following the occurrence of DNA damages or DNA replication stress. Activation of ATR by exogenous DNA damaging agents such as UV leads to phosphorylation of several effector molecules, including Chk1 and H2AX (histone 2A, variant X; NM_002105), inducing the formation of DNA repair foci containing γ-H2AX (phosphorylated H2AX), MDC1 (mediator of DNA damage checkpoint 1), 53BP1 (p53 binding protein 1; NM_001141979), BRCA1 (breast cancer 1), as well as the RPA (replication protein A), 9-1-1 (Rad9-Hus1-Rad1), and Rad17 complexes on the sites of DNA damage [15], [16]. Activation of ATR by Vpr similarly leads to phosphorylation of Chk1 and to the formation of DNA repair foci containing γ-H2AX, 53BP1, RPA, Hus1, Rad17, and BRCA1 [13], [14], [17], [18]. The immediate cause of the activation of ATR following Vpr expression has remained elusive but implicates in part the recruitment by Vpr of the host DDB1 (damage DNA binding protein 1; NM_001923)-CUL4A (cullin 4A; NM_003589) E3 ubiquitin ligase complex via a direct binding to the substrate specificity receptor VPRBP (Vpr-binding protein, also known as DCAF1; NM_014703) [19], [20], [21], [22], [23], [24], [25]. Specifically, RNA interference-mediated depletion of VPRBP or mutations in the hydrophobic leucine-rich core domain of Vpr impaired association to the E3 ligase complex and induction of G2 arrest. In contrast, G2 arrest-defective mutants of Vpr in the C-terminal arginine-rich domain, which maintained their association to the E3 ligase, nevertheless failed to induce G2 arrest [19], [20], [21], [22], [23], [24], [25]. These results indicate that association of Vpr to the E3 ligase complex is required but not sufficient to induce G2 arrest, thus supporting a model in which Vpr would act as a connector between a ubiquitin ligase complex and a yet-unknown cellular protein. We recently provided evidence that Vpr-induced K48-polyubiquitination and proteasomal degradation of this protein(s) would lead to DNA damage/stress, activation of ATR, and ultimately G2 cell cycle arrest [26]. HIV-2 and some species of simian immunodeficiency virus (SIV) encode a paralog of Vpr, called Vpx, which does not induce G2/M arrest but instead counteracts a putative restriction factor expressed in macrophages and dendritic cells that affects infection at a post-entry step [1]. Interestingly, Vpx also interacts with DDB1-CUL4A (VPRBP) via its hydrophobic leucine-rich core domain. This association is required for the inactivation of the restriction factor and probably leads to its proteasomal degradation [27], [28], [29].

The subcellular localization of Vpr and its importance for the induction of G2 arrest has remained a source of controversy. Several investigators reported that Vpr expressed in absence of any other viral proteins primarily localized to the nucleus in a diffuse pattern [30], [31], [32], [33], [34], [35], [36] while others observed a significant accumulation at the nuclear envelope [37], [38], [39], [40], [41], [42]. Of note, Sherman et al. showed that Vpr shuttles between the cytoplasm and nucleoplasm [43]. Moreover, Vpr has been shown to form punctuate structures in the nucleus [17] as well as to induce and co-localize with nuclear membrane herniations [44]. C-terminal mutations impairing G2 arrest did not alter localization of Vpr whereas other mutations, predominantly in the first alpha-helix, impaired both nuclear localization and G2 arrest, implying that nuclear/nuclear-envelope localization of Vpr would be required but not sufficient for this activity [33], [38]. In agreement with this model, Lai et al showed that nuclear punctuate structures formed by Vpr were associated to chromatin and partially co-localized with γ-H2AX, suggesting that Vpr might target host cell DNA and interfere with DNA replication [17]. In contrast, the F34I, V57L, R62P, L68S, and I70S mutations in Vpr caused a re-localization of the protein to the cytoplasm without significantly affecting the induction of G2 arrest [30], [36], [37], [41]. Although inconsistent results were also obtained for some of these mutants [38], these data would suggest instead that Vpr does not induce G2 arrest from the nucleus but from an extra-nuclear compartment.

Therefore, the spatial prerequisites for the induction of Vpr-mediated G2 arrest remain unclear. Additionally, while recruitment of the DDB1-CUL4A (VPRBP) E3 ubiquitin ligase complex has been shown to be critical for G2 arrest, the subcellular compartment where this association occurs and becomes functionally relevant is still unknown. We thus sought to locate the Vpr-VPRBP interaction and to determine the relevance of this localization for the induction of G2 arrest with the goal of furthering our understanding of the mechanism underlying Vpr activation of ATR and providing important information on the potential substrate targeted by Vpr. Herein, we show that Vpr forms nuclear foci that contain VPRBP and that partially co-localize with DNA repair foci components, such as γ-H2AX, RPA32 (replication protein A2, 32kD; NM_002946) and 53BP1. Moreover, we provide evidence that formation of these Vpr nuclear foci constitute a critical early event in the induction of G2 arrest. We also show that Vpr associates to chromatin via its C-terminal domain and that it binds VPRBP on chromatin. Finally, we observed that Vpr foci were highly mobile nuclear bodies. Our results suggest that Vpr recruit the DDB1-CUL4A (VPRBP) E3 ubiquitin ligase complex within mobile nuclear structures to target a chromatin-bound substrate whose ubiquitination and proteolysis would activate ATR and induce G2 arrest.

## Results

### HIV-1 Vpr forms nuclear foci containing VPRBP

**Figure 1:**
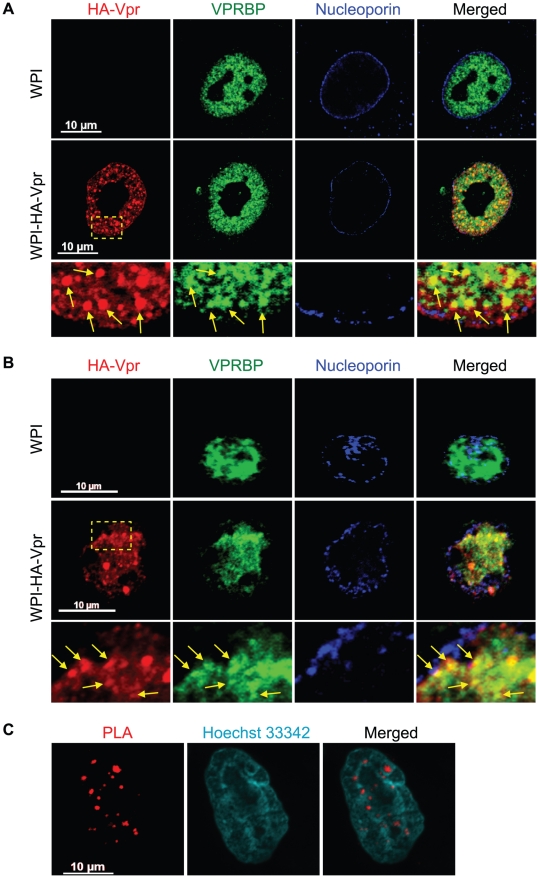
HIV-1 Vpr forms nuclear foci containing VPRBP. **A**) HeLa cells were transduced with lentiviral vectors expressing GFP (WPI) or co-expressing GFP and HA-tagged Vpr (WPI-HA-Vpr) at a multiplicity of infection of 0.5. **B**) Primary activated CD4+ T-lymphocytes were transduced by spinoculation with WPI or WPI-HA-Vpr at a multiplicity of infection of 2.5. For both panels, two days after transduction, cells were fixed, permeabilized, and stained with antibodies against HA (red), nucleoporin (blue) and VPRBP (green). Images were acquired by confocal microscopy with a 63× objective. Images shown are representative of multiple fields. Enlarged (3×) images are shown below panels. Yellow arrows highlight examples of punctuate co-localization. **C**) HeLa cells were transfected with a plasmid expressing HA-Vpr. In situ proximity ligation assay (PLA) was performed on HeLa cells stained with a mouse monoclonal antibody against HA and a rabbit polyclonal antibody against VPRBP. A flurochrome-labeled probe (red) was then used to reveal locations of close proximity between the two proteins. Hoechst 33342 was used to highlight nuclei (cyan). Images were acquired by confocal microscopy with a 63× objective. Images shown are representative of multiple fields.

The interaction between Vpr and VPRBP was previously revealed to be required for the induction of a G2 cell cycle arrest [19], [20], [21], [22], [23], [24], [25]. However, the subcellular localization where this event might take place still remains to be determined. To this end, we performed laser-scanning confocal fluorescence immunohistochemistry to identify the respective subcellular localization and potential co-localization of Vpr and VPRBP. HeLa cells were transduced with a lentiviral vector co-expressing HA-tagged Vpr (HA-Vpr) and GFP or a control lentiviral vector expressing GFP alone. Forty-eight hours after transduction, cells were fixed, permeabilized and stained with antibodies against HA, VPRBP, and nucleoporin. The localization of HA-Vpr was mostly diffuse in the nucleus at standard amplification gain (data not shown). However, when the gain was reduced, we could observe that HA-Vpr formed small circular nuclear structures of variable relative sizes that we refer to thereafter as Vpr nuclear foci (VNF) (Figure 1A). The number of Vpr nuclear foci varied from cell to cell and from experiment to experiment but generally averaged 35 foci (SD±10) per cell. Formation of these foci was not due to the HA tag because we observed that native Vpr could also form nuclear foci (Figure S1A). Endogenous VPRBP was found to be mostly localized to the nucleus in a punctuate pattern (Figure 1A). We observed that HA-Vpr colocalized with endogenous VPRBP in the nucleus. Strikingly, a significant fraction but not all of Vpr nuclear foci co-localized with VPRBP foci, suggesting that Vpr might be able to recruit the E3 ubiquitin ligase complex to these discreet structures. Of note, in presence of Vpr, we also observed some nuclear membrane perturbations reminiscent of the previously described Vpr-induced membrane herniations [44]. Importantly, transduction of activated primary CD4+ T-lymphocytes with a lentiviral vector expressing HA-Vpr also resulted in the formation of Vpr nuclear foci that co-localized with VPRBP (Figure 1B), indicating that these foci are not solely the result of overexpression of Vpr in transformed cell lines but that their formation also occurs in a physiological cellular host of HIV. Infection of HeLa cells with a VSV-G- pseudotyped virus expressing HA-Vpr (HxBru HA-Vpr) also resulted in the formation of Vpr nuclear foci in a minor fraction of cells (Figure S1B). However, the majority of cells displayed a relocalization of HA-Vpr to cytoplasmic compartments (Figure S1B), suggesting that formation of these foci would be a dynamic process, regulated over time during the infection cycle. Indeed, preventing Vpr interaction with Gag and subsequent packaging of the protein into virions using mutations in the p6 domain of Gag (LF/PS) or in Vpr (L23F) [2], [32], resulted in the accumulation of Vpr nuclear foci (Figure S1C). These results provide evidence of the dynamic interplay between Vpr nuclear foci and Gag during infection.

To show that the observed co-localization between Vpr and VPRBP foci was not fortuitous and that Vpr foci truly contained VPRBP, we used an *in situ* proximity ligation assay (PLA) [45]. This assay is based on the ligation of antibody-coupled DNA molecules when these are in close proximity (when secondary antibodies are less than 400 angströms apart). Amplification of ligation products and hybridization with fluorochrome-labelled probes allow the detection of physiological interaction *in situ* without the need to overexpress proteins fused to fluorescent markers. Using this technique, HA-Vpr was found in close proximity to endogenous VPRBP in dense nuclear foci (Figure 1C). We did not observe similar interactions in mock-transfected cells (Figure S2), in cells expressing a Vpr mutant (Q65R) impaired for its interaction with VPRBP [19], [20], [22] (Figure S2), or when any of the primary antibodies where omitted (data not shown). These results therefore suggest that Vpr forms nuclear foci containing VPRBP.

### Vpr nuclear foci partially co-localize with DNA repair foci components

**Figure 2:**
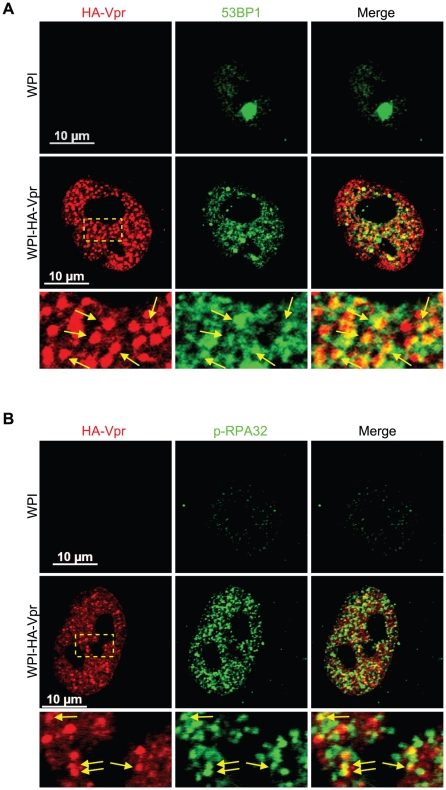
Vpr nuclear foci co-localizes partially with DNA repair foci. HeLa cells were transduced with lentiviral vectors expressing GFP (WPI) or co-expressing GFP and HA-tagged Vpr (WPI-HA-Vpr) at a multiplicity of infection of 0.5. Two days after transduction, cells were fixed, permeabilized, and stained with antibodies against HA (red) and with either rabbit polyclonal antibodies against 53BP1 (green) (**A**) or phospho-RPA32 (green) (**B**). Images were acquired by confocal microscopy. Images shown are representative of multiple fields. Enlarged (3×) images are shown below panels. Yellow arrows highlight examples of punctuate co-localization.

To investigate the nature and composition of these Vpr nuclear foci, we first evaluated whether these would correspond to known well-defined nuclear bodies with similar sizes and numbers. We did not however find any significant co-localization with the canonical nuclear speckle marker SC35 (also known as SFRS2) or with PML (promyelocytic leukemia) bodies (Figure S3). Lai et al. previously reported formation and partial co-localization of Vpr nuclear foci with γ-H2AX [17]. We thus evaluated if the Vpr nuclear foci described herein where the same foci that Lai et al. reported. Interestingly, we observed a partial co-localization between HA-Vpr nuclear foci and 53BP1 (Figure 2A). Indeed, expression of HA-Vpr induced the re-localization of 53BP1 from its sites of residence in the nucleus to DNA repair foci, some of which were positive for HA-Vpr foci. We also observed a partial co-localization between some HA-Vpr nuclear foci and phosphorylated RPA32 (Figure 2B). Similar results were obtained for γ-H2AX (Figure S4A).

### Formation of Vpr nuclear foci represents a critical early event in Vpr-mediated G2 arrest

**Figure 3:**
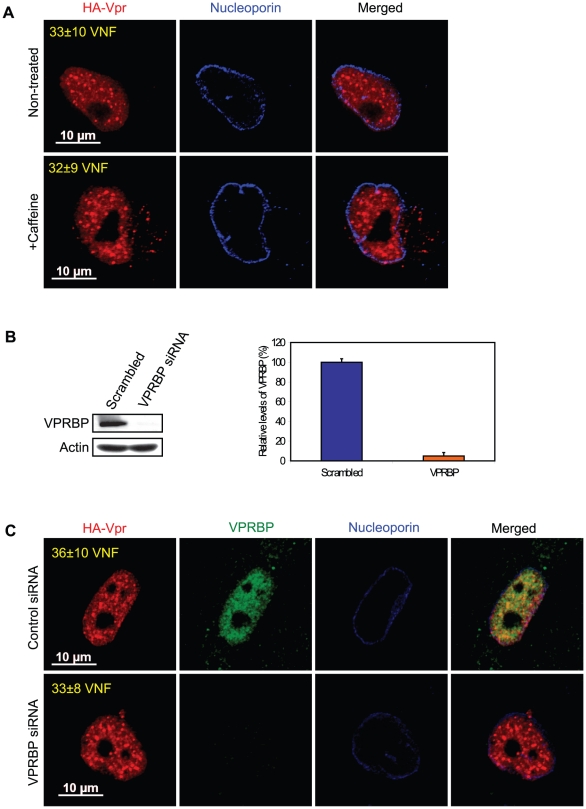
Formation of Vpr nuclear foci is independent of ATR activation and of the recruitment of VPRBP. **A**) HeLa cells were pre-treated with 2.5 mM caffeine for 1 hour and then transduced with lentiviral vectors co-expressing GFP and HA-Vpr (WPI-HA-Vpr) or expressing GFP alone (WPI). One day after transduction, cells were fixed, permeabilized, and stained with antibodies against HA (red) and nucleoporin (blue). Images were acquired by confocal microscopy. Images shown are representative of multiple fields. Averages of the number of Vpr nuclear foci (VNF) per cell and corresponding standard deviations are shown. **B**) HeLa cells were transfected with control scrambled siRNA or siRNA targeting VPRBP. Forty-eight hours after transfection, cells were lysed and expression of VPRBP was monitored by western blot. VPRBP and actin were detected using rabbit polyclonal antibodies. Levels of VPRBP were monitored by computer-assisted densitometry and normalized for actin levels. The means (expressed as percentage relative to levels of VPRBP in scrambled siRNA-transfected cells (100%)) of three independent experiments are depicted in the graph on the right panel. **C**) HeLa cells were transfected with control scrambled siRNA or siRNA targeting VPRBP. Twenty-four hours after transfection, cells were transduced with lentiviral vectors co-expressing GFP and HA-Vpr (WPI-HA-Vpr) or expressing GFP alone (WPI). One day after transduction, cells were fixed, permeabilized, and stained with antibodies against HA (red), nucleoporin (blue) and VPRBP (green). Images were acquired by confocal microscopy. Images shown are representative of multiple fields. Averages of the number of Vpr nuclear foci (VNF) per cell and corresponding standard deviations are shown.

Co-localization of Vpr with components of DNA repair foci suggest that formation of Vpr nuclear foci might represent an early event in the induction of G2 arrest that would be responsible for the generation of DNA replication stress or DNA damage. Conversely, those might simply reflect the re-organization of the nuclear compartment following the activation of the ATR checkpoint by Vpr. To distinguish between these two possibilities, we transduced HeLa cells with a lentiviral vector expressing HA-Vpr and concomitantly treated the cells with caffeine, a non-specific inhibitor of ATR and ATM (ataxia telangiectasia mutated). In these conditions, the addition of caffeine inhibited Vpr-induced cell cycle arrest (data not shown; [12]). However, we did not detect significant changes in the number of Vpr nuclear foci (Figure 3A, 33±10 for non-treated cells vs 32±9 for caffeine-treated cells), suggesting that formation of these foci would take place independently of the activation of ATR. Moreover, consistent with the observation that not all Vpr nuclear foci co-localized with VPRBP (Figure 1A), depletion of VPRBP by siRNA (95%±3.5% knockdown relative to scrambled siRNA) in HeLa cells (Figure 3B) did not significantly alter the number of foci (36±10 for control siRNA vs 33±8 for VPRBP siRNA) (Figure 3C), suggesting that VPRBP is dispensable for the formation of Vpr nuclear foci. Similar results (data not shown) were obtained in a HEK293T monoclonal cell line stably depleted of VPRBP [26]. Moreover, in contrast to its absence of effect on Vpr foci, knockdown of VPRBP abrogated Vpr-induced formation of DNA repair foci containing γ-H2AX and 53BP1 (Figures S4A and S4B). These results indicate that Vpr forms nuclear foci prior to and independently of the activation of ATR and suggest that it is Vpr that recruits VPRBP to these foci and not the inverse.

**Figure 4:**
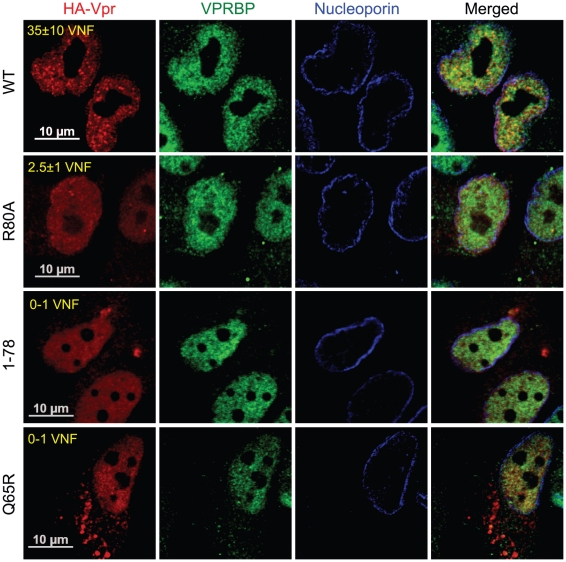
Analysis of the capacity of Vpr mutants to form nuclear foci. HeLa cells were transfected with plasmids expressing HA-tagged Vpr (WT), Vpr (Q65R), Vpr (R80A), and Vpr (1–78). Forty-eight hours after transfection, cells were fixed, permeabilized, and stained with antibodies against HA (red), nucleoporin (blue) and VPRBP (green). Images were acquired by confocal microscopy. Images shown are representative of multiple fields. Averages of the number of Vpr nuclear foci (VNF) per cell and corresponding standard deviations are shown.

To evaluate the potential role of these Vpr nuclear foci in the induction of G2 arrest, we monitored the capacity of several G2 arrest-defective Vpr mutants to form these foci. HeLa cells were transfected with plasmids expressing HA-tagged Vpr mutants and formation of nuclear foci was evaluated by fluorescence immunohistochemistry and confocal microscopy (Figure 4). We found that Vpr (R80A), which still interacts with the E3 ligase but is strongly attenuated for the induction of G2 arrest, was defective for the formation of nuclear foci (2.5±1.1), even though its subcellular localization was nuclear. Deletion of the C-terminus of Vpr (Vpr 1–78), which also maintains the association with the E3 ligase [22] but impairs the induction of G2 arrest [46], similarly resulted in a defect in the formation of nuclear foci (Figure 4). Similar results were also obtained with the C-terminal mutants Vpr (S79A) and Vpr (1–86) (data not shown). Vpr (Q65R), which is unable to associate with the E3 ligase and is consequently defective for G2 arrest, was found to be defective for the formation of nuclear foci and also accumulated in cytoplasmic aggregates. Similar localization phenotypes where observed for Vpr (H71R), a mutant of Vpr also defective for its interaction with VPRBP [21] (data not shown). The results obtained with the Q65R and H71R mutations are in contrast with the siRNA-mediated depletion of VPRBP which did not block the formation of Vpr nuclear foci, suggesting that these mutant proteins might have additional defects besides an impaired interaction with VPRBP (see below). These results thus suggest that the C-terminal domain of Vpr, which is required for the induction of G2 arrest, is also critical for the formation of Vpr nuclear foci.

**Figure 5:**
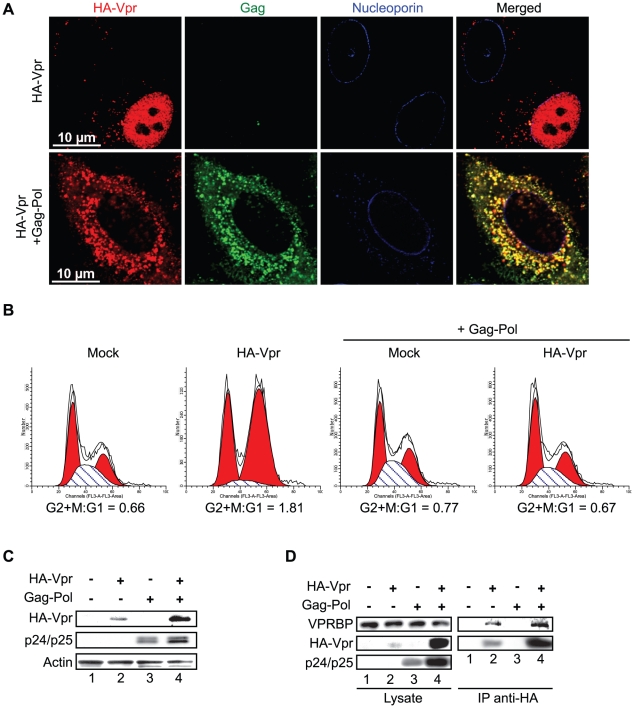
Cytoplasmic sequestration of Vpr abrogates foci formation and G2 arrest. **A**) HeLa cells were co-transfected with the packaging plasmid psPAX2 encoding Gag-Pol, Tat, and Rev and with a HA-Vpr-expressing plasmid or appropriate empty plasmid control. Two days after transfection, cells were fixed, permeabilized, and stained with antibodies against HA (red), nucleoporin (blue) and p24 (green). Images were acquired by confocal microscopy. Images shown are representative of multiple fields. **B**) HEK293T cells were co-transfected with plasmids expressing GFP, HA-Vpr and Gag-Pol (psPAX2) or with an empty plasmid control as indicated. Forty-eight hours after transfection, cell cycle analysis was performed by flow cytometry using propidium iodide staining. Percentages of G1 and G2/M cell populations were determined using the ModFit software. **C**) Expression of HA-Vpr and p24 was monitored by western blot using specific monoclonal antibodies. Actin was detected using a rabbit polyclonal antibody. **D**) HEK293T cells were transfected as in B). Two days after transfection, cells were lysed and subjected to anti-HA immunoprecipitation as described in Materials and Methods. HA-Vpr, p24 or VPRBP levels were evaluated in cell lysates and immunocomplexes. HA-Vpr and p24 were detected using specific monoclonal antibodies. VPRBP was detected using a rabbit polyclonal antibody.

The observation that C-terminal G2 arrest-defective mutants of Vpr are compromised in their capacity to form nuclear foci suggests that these nuclear foci might constitute an important early event in the induction of G2 arrest by Vpr. To directly address this possibility, we first evaluated the functional effect of artificially sequestering Vpr in the cytoplasm by overexpression of Gag-Pol. Co-transfection of HeLa cells with HA-Vpr- and Gag-Pol-expressing plasmids produced a sequestration of HA-Vpr in p24-positive cytoplasmic compartments (Figure 5A). This sequestration abrogated Vpr nuclear foci formation (Figure 5A). Similar results were obtained in HEK293T cells (data not shown). To evaluate the functional effect of this cytoplasmic sequestration of Vpr, HEK293T cells were co-transfected with plasmids expressing HA-Vpr and Gag-Pol or with adequate empty plasmid controls. Two days later, the cell cycle and expression profiles of transfected cells were evaluated by flow cytometry and western blot (Figures 5B and 5C). Expression of HA-Vpr alone produced an accumulation of cells in G2/M (G2+M:G1 = 1.81 vs 0.66 for mock-transfected cells). Interestingly, overexpression of Gag-Pol completely abrogated HA-Vpr-induced G2 arrest (G2+M:G1 = 0.67) in absence of any significant effect on the cell cycle when expressed alone (G2+M:G1 = 0.77). Inhibition of Vpr-induced G2 arrest by overexpression of Gag-pol was dependent on the Gag-Vpr interaction and was not the result of some non-specific effects on the cell cycle since Vpr (L23F), a mutant of Vpr unable to bind the p6 domain of Gag [2], could form nuclear foci (Figure S5A) but was impervious to the effect of Gag-Pol on Vpr nuclear localization (Figure S5B) and induction of G2 arrest (Figure S5C). Although overexpression of Gag-Pol led to a reduction of the affinity between HA-Vpr and endogenous VPRBP, the overall increase in the expression of HA-Vpr resulted in an increase in the levels of Vpr-bound VPRBP (Figure 5D), excluding the possibility that overexpression of Gag-Pol inhibited G2 arrest by preventing the Vpr-VPRBP interaction. The observed inhibition of G2 arrest by overexpression of Gag-Pol is however unlikely to have a significant role at physiological levels of expression given that infection with a wild type virus led to a G2 arrest that was as efficient as the one obtained with a virus unable to relocalize Vpr from the nucleus because of a mutation in the P6 domain of Gag (LF/PS) (Figures S1C and S5D). Overall, these results imply that nuclear localization of Vpr and possibly the formation of nuclear foci would be required for the induction of G2 arrest.

**Figure 6:**
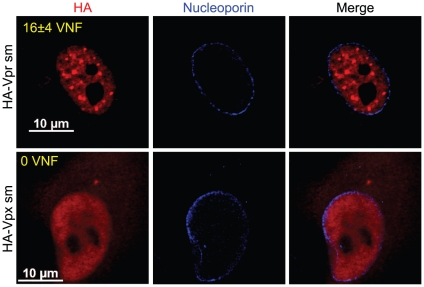
Sooty mangabey Vpr but not Vpx forms nuclear foci. HeLa cells were transfected with plasmids expressing sooty mangabey HA-tagged Vpr (HA-Vpr sm) or Vpx (HA-Vpx sm). Two days after transfection, cells were fixed, permeabilized, and stained with antibodies against HA (red) and nucleoporin (blue). Images were acquired by confocal microscopy. Images shown are representative of multiple fields. Averages of the number of Vpr nuclear foci (VNF) per cell and corresponding standard deviations are shown.

To further show that the formation of these Vpr nuclear foci is critical for the induction of G2 arrest, we evaluated the capacity of SIV Vpr and its paralog Vpx to form these foci. Both of these proteins are able to associate with the E3 ligase complex but in contrast to Vpr, Vpx does not induce G2 arrest but counteract a putative restriction factor in macrophages and dentritic cells [27], [28], [29]. HeLa cells were transfected with plasmids expressing either HA-tagged sooty mangabey Vpr (HA-Vpr sm) or Vpx (HA-Vpx sm). Two days after transfection, cells were fixed, permeabilized, and stained with antibodies against HA and nucleoporin (Figure 6). Consistent with its ability to induce G2 arrest (data not shown and [9]), we found that Vpr sm could accumulate into nuclear foci (16±4 foci per cell) in contrast to the G2-arrest incompetent Vpx that did not form any foci despite being present in the nucleus (Figure 6).

Taken together, these results indicate that formation of Vpr nuclear foci is an early event that is required to induce G2 arrest. These results also indicate that nuclear localization of Vpr is not sufficient to induce formation of foci.

### Vpr oligomerization is not sufficient to induce foci formation

**Figure 7:**
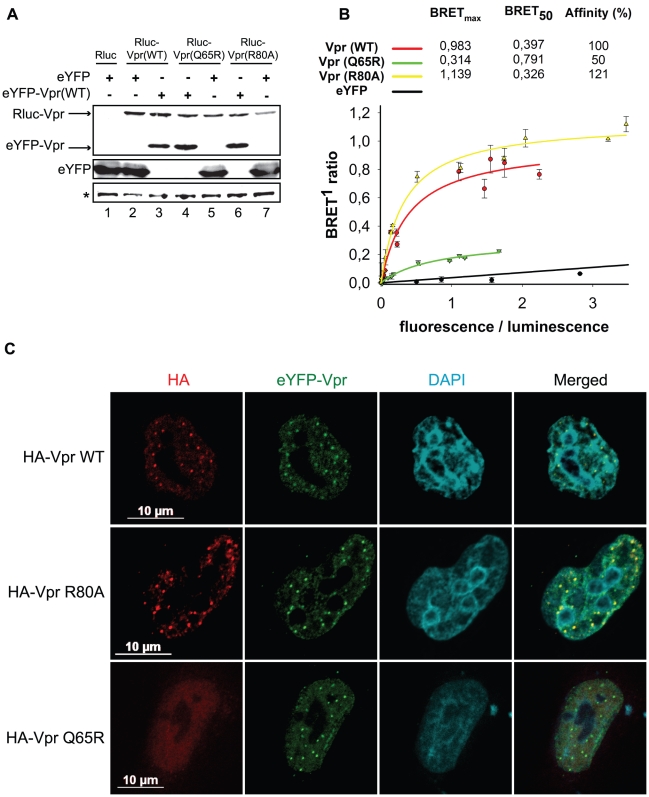
Analysis of the self-affinity of wild type Vpr and mutants. **A**) HEK293T cells were either co-transfected with plasmids expressing Rluc and eYFP or co-transfected with plasmids expressing Rluc-Vpr (WT) or Rluc-Vpr (Q65R), or Rluc-Vpr (R80A) and eYFP-Vpr (WT) or eYFP. Two days later, cell lysates were resolved by SDS-PAGE and protein expression was determined by western blot using rabbit polyclonal antibodies directed against Vpr and GFP. A non-specific band, depicted by the asterisk, was used as loading control. **B**) BRET saturation assays were performed with live HEK293T cells. A plasmid expressing Rluc-Vpr (WT), Rluc-Vpr (Q65R) or Rluc-Vpr (R80A) (BRET donor) was co-transfected with increasing concentration of a plasmid expressing eYFP-Vpr (BRET acceptor) or eYFP (non-specific control). Forty-eight hours post-transfection, energy transfer was initiated by addition of the cell-permeable *renilla* luciferase substrate coelenterazine H. Donor saturation curves were obtained by measuring BRET in presence of a fixed quantity of donor and increasing amounts of acceptor. The x-axis shows the ratio between the fluorescence (520 nm) of the acceptor (YFP-YFP_0_, where YFP_0_ is the fluorescence value in cells expressing the BRET donor alone) and the luminescence (475 nm) of the donor. BRET ratios (y-axis) were calculated as described in Materials and Methods. BRET_max_ is the maximal BRET signal reached at saturation. BRET_50_, which represents the concentration (fluorescence/luminescence) of acceptor giving 50% of BRET_max_, is a measure of the relative affinity of each fusion protein. Self-affinities relative to wild type are depicted in the graph. Curves shown represent the means ± standard deviations of results from one representative experiment performed in duplicate. The curves were generated by non-linear regression, in which a single binding site was assumed using the Sigma Plot software v.10. **C**) HeLa cells were co-transfected with plasmids expressing eYFP-Vpr and HA-Vpr WT, R80A, or Q65R. Two days after transfection, cells were fixed, permeabilized, and stained with antibodies against HA (red). EYFP-Vpr was detected by direct fluorescence (green) and DAPI (4,6-diamidino-2-phenylindole) was used to highlight the nuclei (cyan). Images were acquired by confocal microscopy. Images shown are representative of multiple fields.

Given that these foci constitute an early event in the induction of G2 arrest, we sought to determine how they would form. These foci are likely the results of a local observable accumulation of Vpr either through oligomerization of the protein or following its recruitment by a locally abundant tethering factor. To distinguish between these two possibilities, we first monitored the dimerization efficiency of the Vpr mutants Q65R and R80A, which are defective for foci formation. HEK293T cells were co-transfected with plasmids expressing enhanced yellow fluorescence protein (eYFP) fused to the N-terminus of wild type Vpr and *renilla* luciferase (Rluc) fused to the N-terminus of wild type Vpr and mutants. Two days after transfection, self-affinity was assessed by bioluminescence resonance energy transfer (BRET). Figure 7A reveals that all Vpr fusion proteins were efficiently expressed. In this system, we observed a specific energy transfer between eYFP-Vpr (WT) and Rluc-Vpr (WT) (Figure 7B). The maximum energy transfer at saturation (BRET_max_) was 0.983 and the concentration of acceptor at 50% of BRET_max_ (BRET_50_) was 0.397. In contrast, co-expression of eYFP and Rluc-Vpr did not lead to any significant energy transfer, demonstrating the specificity of the eYFP-Vpr/Rluc-Vpr interaction. The Q65R mutant, showed a significant decrease in its affinity for wild type eYFP-Vpr (BRET_50_ = 0.791, 50% self-affinity) as well as a drastic decrease in BRET_max_ (0.314 for Q65R vs 0.983 for wild type Vpr), suggesting that in addition to a reduction in dimerization efficiency, formation of higher-order complexes (multimerization) would also be synergistically decreased. In contrast, the R80A mutant displayed an affinity for wild type Rluc-Vpr that was at least comparable to wild type Vpr (BRET_50_ = 0.326, 121% self-affinity relative to wild type). Similar results were obtained when eYFP-Vpr R80A and Rluc-Vpr R80A were co-expressed (data not shown). Thus, these results suggest that the ability of Vpr to oligomerize does not directly correlate with nuclear foci formation and does not explain the defect in foci formation observed in the context of C-terminal mutants. To determine if oligomerization of Vpr could still be involved in formation of Vpr nuclear foci, we performed trans-complementation experiments in HeLa cells and monitored formation of Vpr foci by immunofluorescence confocal microscopy. Trans-complementation of HA-Vpr R80A with eYFP-Vpr could rescue the defective phenotype of the R80A mutant by re-localizing the protein to eYFP-Vpr foci (Figure 7C). In contrast, eYFP-Vpr was unable to re-localize the HA-tagged Q65R mutant (Figure 7C), suggesting that oligomerization of Vpr, although not sufficient to induce formation of Vpr foci, may however contribute to the process to some degree.

### Association of Vpr to chromatin correlates with formation of nuclear foci

**Figure 8:**
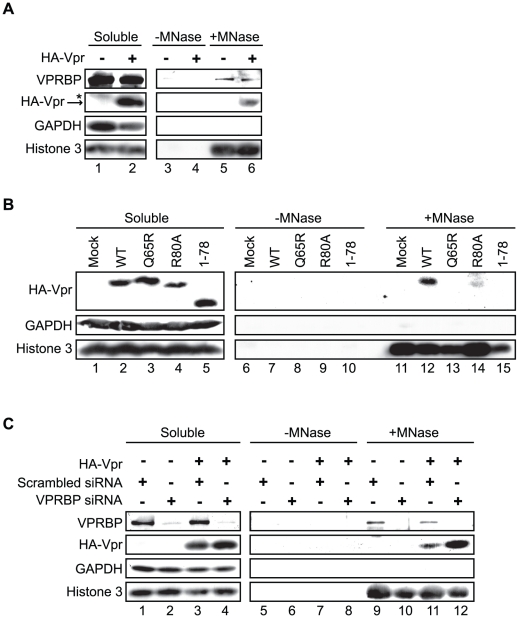
Association of Vpr with chromatin correlates with the formation of nuclear foci. **A**) HeLa cells were transfected with plasmids expressing HA-tagged Vpr (WT) or an empty plasmid used as negative control. Forty-eight hours after transfection, cells were harvested and lysed with 0.5% Triton X-100. The soluble fraction was used as input control (Soluble). Insoluble debris containing chromatin was treated with microccocal nuclease (+MNase) or with buffer alone (−MNase). The resulting solubilized fractions and input controls were resolved by SDS-PAGE and analyzed by western blot. Specific monoclonal antibodies were used to detect GAPDH (cytoplasmic marker) and HA-Vpr. Histone 3 (chromatin marker) and VPRBP were detected using rabbit polyclonal antibodies. * Denotes a non-specific band detected with the anti-HA antibody. **B**) HeLa cells were transfected with plasmids expressing HA-tagged Vpr (WT), Vpr (Q65R), Vpr (R80A), and Vpr (1–78). Cell extracts were processed and analysed as in A). **C**) HeLa cells were first transfected with scrambled siRNA or siRNA targeting VPRBP. Twenty-four hours after transfection, cells were transfected with a plasmid expressing HA-Vpr (WT) or an empty plasmid as negative control. Cell extracts were processed and analyzed as in A).

Since oligomerization does not fully account for the ability of Vpr to form foci, Vpr could thus be tethered to specific sites by a cellular co-factor. Co-localization of Vpr nuclear foci with chromatin-bound factors detected at DNA repair sites suggests that this tethering co-factor could be a chromatin-bound protein or structure or DNA itself. To assess this possibility, HeLa cells were first transiently transfected with an empty plasmid or a plasmid expressing HA-Vpr and cells were lysed with 0.5% Triton X-100, resulting in the release of soluble proteins. Treatment of Triton-insoluble cellular material, including chromatin, with microccocal nuclease resulted in the solubilization of chromatin-bound cellular proteins including RPA70 (replication protein A1, 70 kDa) (data not shown) and histone 3 (Figure 8A). These proteins were not detected when cell extracts were incubated in buffer without microccocal nuclease. Importantly, chromatin extracts were not contaminated with cytoplasmic proteins as revealed by the absence of GAPDH (glyceraldehyde-3-phosphate dehydrogenase) (Figure 8A). Using this system, we found that a fraction of HA-Vpr was released in extracts treated by microccocal nuclease but not with buffer alone, indicating that Vpr associates with chromatin directly or indirectly via other proteins (Figure 8A). A specific association of a fraction of endogenous VPRBP with chromatin was also observed in presence and in absence of Vpr (Figure 8A). To determine whether the defects of foci formation observed with C-terminal mutants of Vpr would correlate with a defect in chromatin association, we analyzed the capacity of several Vpr mutants to associate to chromatin in HeLa cells. Interestingly, both Vpr (R80A) and a C-terminal deletion mutant (Vpr 1–78) showed a drastic reduction in their association to chromatin (Figure 8B). Of note, Vpr (Q65R) (Figure 8B) and Vpr (H71R) (data not shown) also failed to associate with chromatin, possibly explaining their unexpected incapacity to form foci. In contrast, knockdown of VPRBP did not significantly alter the affinity of Vpr for chromatin (Figure 8C), suggesting that VPRBP does not contribute to this association and that the absence of chromatin association with the Q65R and H71R mutants is not due to its impaired binding to VPRBP. Therefore, the ability of Vpr to form foci correlates with its ability to associate with chromatin.

### Vpr and VPRBP interact on chromatin

**Figure 9:**
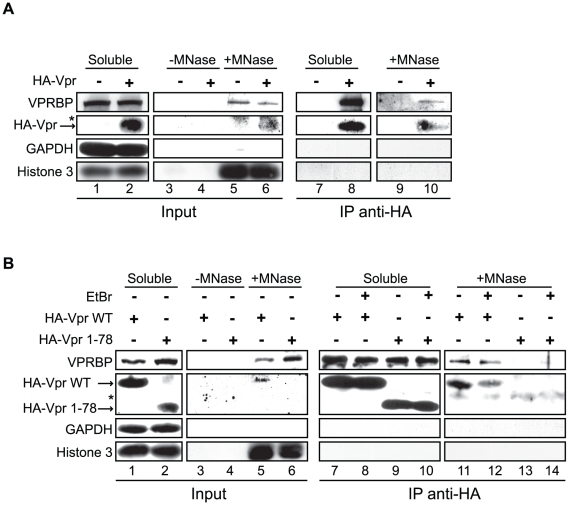
Vpr and VPRBP associate on chromatin. **A**) HeLa cells were transfected with a plasmid expressing HA-tagged Vpr (WT) or an empty plasmid used as negative control. Soluble and chromatin-bound fractions were isolated and were subjected to anti-HA immunoprecipitation as described in Materials and Methods. **B**) HeLa cells were transfected with a plasmid expressing HA-tagged Vpr (WT) or Vpr (1–78). Soluble and chromatin-bound fractions were isolated and subjected to anti-HA immunoprecipitation as described in Materials and Methods. Half of the immunoprecipitations (lanes 8, 10, 12, and 14) were conducted in presence of 25 µg/ml ethidium bromide (EtBr) to displace proteins from DNA. For both panels, input controls and immunoprecipitates were resolved by SDS-PAGE and analyzed by western blot. Specific monoclonal antibodies were used to detect GAPDH (cytoplasmic marker) and HA-Vpr. Histone 3 (chromatin marker) and VPRBP were detected using rabbit polyclonal antibodies. * Denotes a non-specific band detected with the anti-HA antibody.

Co-localization of Vpr nuclear foci with VPRBP and the association of both proteins to chromatin suggest that they might interact on chromatin. To evaluate this possibility, we transfected HeLa cells with an empty plasmid or a plasmid expressing HA-Vpr and performed anti-HA immunoprecipitations on proteins released from chromatin by microccocal nuclease (Figure 9A). Interestingly, we could detect co-immunoprecipitation of endogenous VPRBP specifically in cells extracts containing HA-Vpr, in the soluble fraction as well as in the chromatin fraction (Figure 9A). Deletion of the C-terminal domain of Vpr abrogated its interaction with VPRBP on chromatin but not in the soluble fraction (Figure 9B), demonstrating the specificity of these interactions. These data suggest that Vpr interacts with VPRBP on chromatin. Importantly, histone 3 did not co-immunoprecipitate with HA-Vpr in the chromatin fraction (Figure 9A). Moreover, treatment with high concentrations of ethidium bromide during the immunoprecipitation, a treatment that displace proteins from DNA [47], did not disrupt the Vpr-VPRBP interaction in the soluble fraction as well as on chromatin (Figure 9B), thus excluding the possibility that the observed Vpr-VPRBP interaction was mediated by incompletely digested chromatin fragments.

### Vpr foci are highly mobile long-lasting nuclear bodies

**Figure 10:**
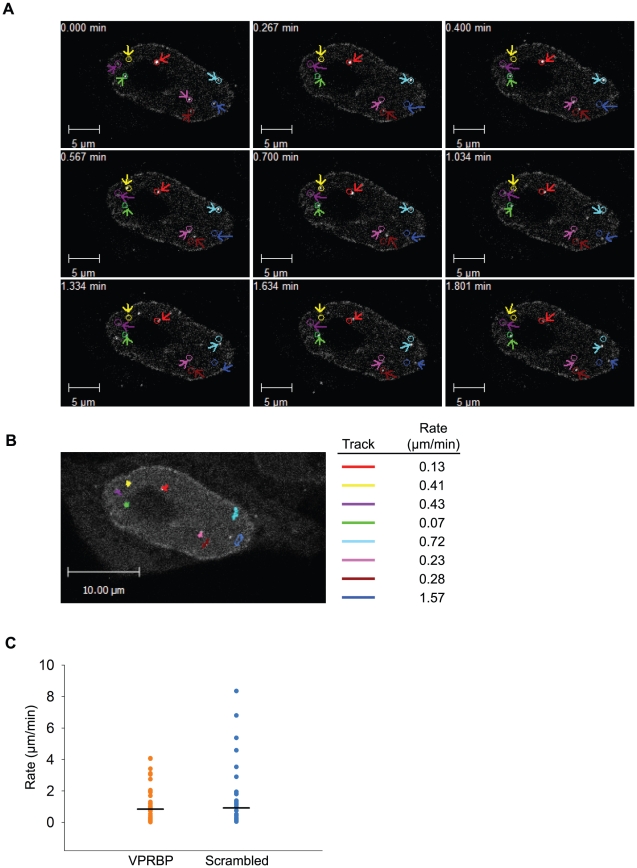
Vpr nuclear foci are mobile nuclear bodies. **A**) HeLa cells were transfected with a plasmid expressing eYFP-Vpr WT. Two days after transfection, the location of eYFP-Vpr was monitored by time-lapse confocal microscopy in living cells. Images were acquired with a 63× objective at intervals of 2 seconds for two minutes. Representative images taken at time points spanning the period of acquisition are shown. The initial positions of Vpr foci are depicted by colored circles. The actual positions of foci are indicated with colored arrows. **B**) Vpr foci in images acquired in A) were tracked using the Volocity software v.5.2.1. Movement tracks of foci are depicted in colors on the picture. Rates of displacement for each focus are indicated at the right of the picture. Please note that some foci could not be tracked for the full time of acquisition because they were migrating out of the focal plane. **C**) HeLa cells were first transfected with scrambled siRNA or siRNA targeting VPRBP. Twenty-four hours after transfection, cells were transfected with a plasmid expressing eYFP-Vpr. The location of eYFP-Vpr foci was determined as in A) and rates of displacement were calculated as in B). Rates of displacement of individual foci are shown in a dot plot. Averages of displacement rates are indicated as horizontal bars.

Nuclear bodies stably or transiently associating with chromatin are generally dynamic structures, either in mobility or in stability. For instance, PML bodies display varying levels of mobility in the nucleus. Conversely, DNA repair foci show limited mobility but can rapidly form in response to genotoxic stress and can disassemble following checkpoint recovery [48], [49], [50]. To investigate the possible dynamic nature of Vpr nuclear foci, we performed time-lapse confocal microscopy in living HeLa cells expressing eYFP-Vpr. Strikingly, observation of eYFP-Vpr foci for two minutes (at two-second intervals) revealed that these were highly mobile structures (Figure 10A; Videos S1 and S2). Software-assisted tracking of over fifty Vpr foci (Figure 10B and data not shown) revealed rates of displacement ranging from 0.05 µm/min to 8.30 µm/min for an average of 0.73 µm/min (SD = 1.00 µm/min; median = 0.30 µm/min). The mobility of Vpr foci was not dependent on the presence of VPRBP since its knockdown (Figure 10C) did not significantly alter their dynamic behavior (average rate of displacement of 0.85 µm/min for VPRBP siRNA vs 0.92 µm/min for scrambled siRNA; *P* = 0.78). Because some eYFP-Vpr foci seemingly appeared and disappeared during the course of these observations, we performed time-lapse spinning-disk microscopy analyses to evaluate whether these foci were translating in and out of the focal plane or instead assembling and disassembling. Tracking of eYFP-Vpr nuclear foci for 15 minutes at intervals of 5 seconds highlighted translational movements in the three axes (Figure S6A). Moreover, these analyses did not reveal any apparition or disappearance of nuclear foci (data not shown), suggesting that these are structurally stable. Similar results were obtained from observations over longer periods of time (30 minutes). Monitoring of the mean fluorescence of eYFP-Vpr in foci showed relatively stable signal over time (Figure S6A, right panel). Some transient fluctuations in fluorescence were however detected. To determine if these fluctuations could be the result of quick exchange of Vpr molecules in and out of nuclear foci, we performed fluorescence recovery after photobleaching (FRAP) analyses on eYFP-Vpr foci (Figure S6B). Photobleaching of eYFP-Vpr foci did not however lead to any fluorescence recovery even after an extensive period of time (350 seconds), suggesting that the inter-exchange of Vpr molecules is minimal.

Overall, our results suggest that Vpr would associate to chromatin-bound nuclear foci via its C-terminus. These would serve as a mobile scaffold to recruit the DDB1-CUL4A (VPRBP) E3 ubiquitin ligase to induce the ubiquitination and degradation of a chromatin-bound substrate, resulting in DNA damage or replication stress.

## Discussion

Our results show that Vpr mainly localizes to the nucleus in transformed epithelial cells, such as HeLa and HEK293T cells, as well as in primary CD4+ T-lymphocytes (Figure 1 and data not shown). We noticed that the localization of Vpr in HeLa cells closely resembles that observed in primary CD4+ T-lymphocytes, prompting us to select this cellular model for most of our study. Moreover, we found that ectopically expressed HA-tagged Vpr had a subcellular localization similar to that of the native protein (Figure S1A). In infected cells, the nuclear localization of Vpr appears transient because Gag interacts with Vpr to package the protein into assembling viral particles (Figures S1B and S1C). Our localization data show that Vpr can form nuclear punctuate structure that we termed Vpr nuclear foci (Figure 1), as was reported previously by Lai and colleagues [17]. It is noteworthy that these foci are not readily apparent and require careful calibration of gain to be observed (data not shown). Importantly, we observed a strong co-localization of Vpr with VPRBP in the nucleus, particularly in these foci. *In situ* proximity ligation assays confirmed the close proximity of the two proteins in these foci (Figure 1C), suggesting that Vpr interacts with the E3 ubiquitin ligase at the levels of these punctuate structures. In contrast to the observations of other investigators [37], [38], [39], [40], [41], [42], we did not observe a significant accumulation of Vpr at the nuclear membrane in these cell types. Several technical reasons might explain these discrepancies, including cell types, levels of expression, fixation and permeabilization conditions, or the tag used. Indeed, we did observe an enrichment of eYFP-Vpr at the nuclear membrane of Hela cells (Videos S1 and S2).

We obtained several lines of evidence demonstrating that Vpr nuclear foci are involved in Vpr-mediated G2 arrest. First, we observed a partial co-localization between these foci and RPA32, 53BP1 and γ-H2AX, which are usually detected at DNA repair sites (Figures 2 and S4). Similar results were obtained by Lai and colleagues with γ-H2AX [17]. Secondly, C-terminal mutants of Vpr defective for G2 arrest failed to induce formation of Vpr foci despite their nuclear localization (Figure 4). Thirdly, cytoplasmic sequestration of Vpr by overexpression of Gag inhibited G2 arrest as well as foci formation (Figure 5). Fourthly, only Vpr from sooty mangabey SIV but not its G2 arrest-defective paralog Vpx was able to form these foci (Figure 6). Lastly, the reduced number of foci formed by sooty mangabey Vpr in comparison to HIV-1 Vpr correlated with reduced G2 arrest activity in human cells (data not shown and [9]). All these results suggest that formation of foci is linked to G2 arrest. Moreover, these results also suggest that nuclear localization of Vpr is required but not sufficient to induce formation of these foci. Our results and conclusions are in contrast with previous reports, including one of ours, describing cytoplasmic mutants of Vpr that retain their G2 arrest activity [30], [36], [37], [41]. We had reported over a decade ago that the V57L and R62P mutations induced the relocalization of Vpr to the cytoplasm, while these mutants were still able to induce G2 arrest [36]. However, careful re-examination of the localization of these mutants showed that both mutants could localize to the nucleus to some degree. While, the V57L mutant had a reduced capacity to form foci, the R62P mutant was completely defective for foci formation (Figure S7A). The reduced capacity of V57L mutant and the defect of the R62P mutant in foci formation correlated, respectively, with attenuation and abrogation of G2 arrest (Figure S7B). These differences between our present localization data and our previously published results can probably be explained by improved imaging sensitivity, whereas the discrepancies in G2 arrest activity are unclear. Nevertheless, these results highlight an important technical limitation in these types of localization experiments: lack of detection in a subcellular compartment does not necessarily indicate an absence of protein.

Correlation between G2 arrest and formation of Vpr nuclear foci implies that the formation of these foci could either be an early event leading to G2 arrest or could be a consequence of this G2 arrest. We observed that treatment with the ATR/ATM inhibitor caffeine (Figure 3A) did not abrogate formation of Vpr foci, thus indicating that these foci likely constitute an early event in the induction of G2 arrest by Vpr. In fact, formation of Vpr foci was not affected by an almost complete knockdown of VPRBP suggesting that their formation is independent of the recruitment of the E3 ligase complex and would therefore precede ubiquitination and degradation of the putative G2 arrest substrate (Figures 3B and S4). In contrast, we found that the Q65R mutant of Vpr was unable to form foci. In addition to a reduced affinity for VPRBP [19], [20], [22], this mutation also leads to other defects including accumulation of Vpr in the cytoplasm (Figure 4), reduced dimerization efficiency (Figure 7), and absence of binding to chromatin (Figure 8B), indicating that the Q65R mutation has pleiotropic effects on the functions of Vpr. Yet, this mutation did not prevent efficient packaging of Vpr into virions [12]. Cautions should thus be used when interpreting results obtained with this mutant. Despite these pleiotropic defects, we cannot completely exclude the possibility that, in addition to the C-terminal domain, binding to VPRBP would also contribute to foci formation and chromatin association.

Given that Vpr foci containing VPRBP partially co-localize with chromatin-bound protein such as RPA32 and that Vpr associates with DNA *in vitro* [51] and *in vivo* (Figure 8A and [17]), we propose that Vpr might be able to target a chromatin-bound cellular factor. In support of this hypothesis, Lai et al. showed that *in situ* nuclease treatment of Vpr-expressing cells eliminates Vpr nuclear foci [17], suggesting that Vpr nuclear foci are anchored to chromatin. Deletion of the C-terminal domain of Vpr drastically reduced foci formation (Figure 4) and its chromatin association (Figure 8B). Similar results were obtained by Lai and colleagues [17]. Moreover, mutation of the arginine at position 80 did not affect direct binding to nucleic acids *in vitro* [51] but nevertheless impaired association to chromatin *in vivo* (Figure 8B), implying that a cellular factor rather than a direct binding to DNA would be implicated in association to chromatin. This cellular factor does not appear to be VPRBP since its knockdown did not significantly reduce the binding of Vpr to chromatin (Figure 8C). Moreover, we also observed protein-protein interaction between Vpr and VPRBP on chromatin (Figure 9), suggesting that Vpr would be able to recruit the E3 ligase DDB1-CUL4A (VPRBP) onto chromatin.

Strikingly, analysis of Vpr nuclear foci by time-lapse microscopy (Figures 10 and S5, Videos S1 and S2) revealed that these foci moved rapidly in the nucleus (average of 0.73 µm/min). As a comparison, passive diffusion of chromatin-bound DNA repair foci was estimated at 1–2 µm per 6 hours [52]. These results suggest that instead of stably interacting with chromatin, Vpr nuclear foci would do so in a dynamic manner, allowing movement of the foci along chromatin strands. One possible model to integrate all our results is that Vpr could interact with its putative substrate via its C-terminus in these chromatin-bound nuclear foci and could recruit the DDB1-CUL4A(VPRBP) E3 ligase to degrade the substrate, thus preventing its potential role in DNA replication or DNA repair. This model implies that Vpr would initially require binding with the substrate to localize in these nuclear bodies and that the subsequent degradation of this substrate would not exclude Vpr from these structures nor would it disrupt them. Another possibility is that Vpr would interact with a nuclear foci-associated co-factor via its C-terminus and would utilize these mobile structures to scan chromatin for its putative substrate. This second model requires that either Vpr possesses an additional functional domain mediating the interaction with the substrate or that Vpr targets VPRBP's own natural substrates. Irrespective of the above models, as was recently documented, the substrate would be covalently modified with classical K48-linked polyubiquitin chains in a DDB1-CUL4A (VRPBP)-dependent manner and degraded by the proteasome [26]. Moreover, multiple units of the putative substrate/co-factor are probably required in these nuclear bodies in order for Vpr to accumulate in these structures. Even though Vpr multimerization was shown to occur in these foci (Figure 7), it is unlikely that it would play a major role in this process given that the L23F mutation was previously shown to block dimerization [40], [53] but did not significantly affect foci formation and induction of G2 arrest (Figure S5). Similar conclusions were also previously obtained with the I70S mutation which was shown to block dimerization without affecting the induction of G2 arrest [30]. It however remains unclear whether Vpr would bind VPRBP before or after localizing to these foci, particularly when considering the important level of interaction observed in the Triton-soluble fraction (Figure 9). Moreover, the significance of the partial co-localization observed between Vpr and DNA repair foci containing RPA32, 53BP1 and γ-H2AX (Figures 2 and S4) is also unclear. On one hand it could mean that degradation of the chromatin-bound substrate would induce DNA damage or DNA replication stress *in situ* and that this partial co-localization would be explained by the high mobility of Vpr foci. On the other hand, we cannot exclude the possibility that degradation of the substrate could induce global genomic instability and that this partial co-localization would only be fortuitous.

Overall, our results show that Vpr forms highly mobile nuclear foci containing VPRBP and demonstrate that formation of these structures constitutes a critical early event in the induction of DNA damage/stress and G2 arrest by Vpr. The characterization of these chromatin-bound nuclear foci hijacked by Vpr will likely contribute to better delineate the mechanism by which Vpr activates ATR and induces G2 arrest. Importantly, our results further suggest that the putative cellular substrate targeted by Vpr is likely to be a chromatin-associated protein.

## Materials and Methods

### Ethics statement

Peripheral blood samples were obtained from adult donors who gave written informed consent under research protocols approved by the research ethics review board of the Institut de recherches cliniques de Montreal.

### Cells, antibodies, and other reagents

HeLa and HEK293T cells were cultured as previously described [54]. Primary CD4+ T-lymphocytes were isolated and cultured as previously described [26]. The development of the HEK293T cell line stably depleted of VPRBP was described previously [26]. Caffeine and DAPI (4′,6-Diamidino-2-phenylindole) were purchased from Sigma-Aldrich (St. Louis, MO, USA). SiRNA targeting VPRBP (siGenome SMARTpool M-021119-00) and scrambled control siRNA (non-targeting siRNA #2) were obtained from Dharmacon (Chicago, IL, USA). The anti-HA (clone 12CA5) and anti-p24 (catalog no. HB9725) monoclonal antibodies were produced from hybridomas obtained from the American Type Culture Collection (Manassas, VA, USA). The monoclonal antibody against Vpr (clone 8D1) was a kind gift of Dr Y. Ishizaka (International Medical Center of Japan, Tokyo, Japan) [55]. The following commercially available antibodies were used: mouse anti-nucleoporin (Abcam, Cambridge, MA, USA), mouse anti-RPA70 (Abcam), rabbit anti-53BP1 (Abcam), rabbit anti-GAPDH (Cell Signaling Technology, Danvers, MA, USA), rabbit anti-H3 antibodies (Abcam) rabbit anti-phospho RPA32 (S4/S8) (Bethyl Laboratories, Montgomery, TX, USA), rabbit anti-VPRBP (Accurate Chemical and Scientific Corporation, Westbury, NY, USA), rabbit anti-actin (Sigma-Aldrich, St. Louis, MO, USA), mouse anti-phosphoS139-H2AX (clone JBW301)(Upstate, Millipore, Billerica, MA, USA), mouse FITC-conjugated anti-p24 (clone KC57, Beckman Coulter Canada, Mississauga, Ontario, Canada), mouse anti-SC35 (Sigma-Aldrich), and mouse anti-PML (Santa Cruz Biotechnology, Santa Cruz, CA, USA). All fluorochrome-conjugated secondary antibodies were obtained from Molecular Probes (Invitrogen, San Diego, CA, USA).

### Plasmid construction

SVCMV-Vpr (WT), SVCMV-Vpr (L23F), SVCMV-HA-Vpr (WT), SVCMV-HA-Vpr (V57L), SVCMV-HA-Vpr (R62P), SVCMV-HA-Vpr (Q65R), SVCMV-HA-Vpr (H71R), SVCMV-HA-Vpr (R80A), SVCMV-HA-Vpr (S79A), SVCMV-HA-Vpr (1–86), SVCMV-HA-Vpr (1–78), and SVCMV-VSV-G were previously described or were constructed by PCR as previously described [19], [32], [46]. Plasmids pCDNA3.1_eYFP-MCS(MB) and pCDNA3.1_Rluc-MCS(MB) for the expression of eYFP and renilla luciferease (Rluc) N-terminal fusion proteins were kind gifts of M. Baril and D. Lamarre [56]. Wild type Vpr was amplified by PCR from SVCMV-HA-Vpr (WT) and subcloned into pCDNA3.1_eYFP-MCS(MB) and pCDNA3.1_Rluc-MCS(MB) to generate respectively pCDNA3.1-eYFP-Vpr(WT) and pCDNA3.1-Rluc-Vpr (WT). Vpr (R80A) and Vpr (Q65R) were subcloned into pCDNA3.1_Rluc-MCS(MB) to generate pCDNA3.1-Rluc-Vpr (R80A) and pCDNA3.1-Rluc-Vpr (Q65R) using the same strategy. The lentiviral vector pWPI as well as the packaging plasmid psPAX2 expressing Gag-Pol, Tat and Rev were obtained from Dr. D. Trono (School of Life Sciences, Swiss Institute of Technology, Lausanne, Switzerland). The lentiviral vector pWPI-HA-Vpr (WT) transducing HA-tagged Vpr and GFP was generated from the parental vector pWPI using a strategy described previously [19]. The plasmids expressing sooty mangabey HA-tagged Vpr and Vpx were obtained from S. Benichou (Institut Cochin, Paris, France) [4]. The infectious molecular clones HxBru (Vpr-), HxBru (HA-Vpr), and HxBru Vpr L23F, were described previously [26], [32], [57]. The HxBru VprWT LF/PS molecular clone with mutations (L44P, F45S) in the p6 domain of Gag disrupting interaction with Vpr was described previously [58].

### Production and titration of viruses and lentiviral vectors

The production and titration of VSV-G-pseudotyped HIV particles and lentiviral vectors were performed as described previously [19], [46].

### Transfection, transduction and infection

HeLa cells were transfected using the Lipofectamine 2000 reagent (Invitrogen Canada, Burlington, Ontario, Canada) according to the manufacturer's instructions. HEK293T cells were transfected by a standard calcium phosphate precipitation protocol. SiRNA were transfected using Lipofectamine RNAi Max (Invitrogen Canada, Burlington, Ontario, Canada), according to the manufacturer's instructions. HeLa cells were transduced with the lentiviral vectors WPI and WPI-HA-Vpr in presence of 8µg/ml polybrene at a multiplicity of infection of 0.5 to 2.5, as indicated for each experiment. Primary CD4+ T-lymphocytes were transduced by spinoculation at a multiplicity of infection of 1. Briefly, cells were mixed with lentiviral vector particles in presence of 8µg/ml polybrene and centrifuged for 2 hours at 1200g. HeLa cells were infected, in presence of 8 µg/ml polybrene, with VSV-G-pseudotyped HIV-1 viruses at a concentration of 100 cpm/cell or at a MOI of 1.0, as indicated for each experiment.

### Fluorescence microscopy and live-cell imaging

Fifty thousand HeLa cells were seeded on cover slips in 24-well plates. Cells were transfected, transduced, or infected as indicated for each experiment. Two days later, cells were processed for fluorescence immunohistochemistry and laser-scanning confocal microscopy as previously described [59]. For analysis of CD4+ primary T-lymphocytes, 5×10^5^ cells were first adhered on poly-Lysine-treated coverslips for two hours in PBS and then processed as described [59]. Quantification of Vpr nuclear foci was performed in at least 30 randomly selected cells by manual counting. Time-lapse confocal microscopy was performed on living cells in a PeCON environmental chamber maintained at 37°C and 5% CO_2_. Images were acquired using a Zeiss LSM 710 system with the ZEN 2009 software. Spinning-disk confocal microscopy was performed on living cells using a Quorum WaveFX-X1 spinning-disc confocal system (Quorum Technologies Inc, Guelph, Ontario, Canada). Cells were maintained at 37°C in 5% CO_2_ in a Live Cell Instruments Chamlide TC environmental chamber. Images were acquired with a Hamamatsu ImagEM C9100-13 camera using the Metamorph software. FRAP (fluorescence recovery after photobleaching) experiments were conducted using the Quorum WaveFX-X1 spinning-disc confocal system equipped with a Photonic Instruments Mosaic 405 nm laser. Images were processed using AxioVision v.4.7. Videos were generated with the ZEN 2009 software. Software-assisted fluorescence quantification and tracking of Vpr foci was performed with the Volocity software v.5.2.1. Statistical analysis was performed using Sigma Plot software v.10.

### *In situ* proximity ligation assay

*In situ* proximity ligation assays (PLA) were performed using the Duolink kit 613 (Olink bioscience, Uppsala, Sweden). Briefly, HeLa cells were transfected with a plasmid encoding HA-Vpr or an empty plasmid as negative control. At 48h post-transfection, the cells were cytospun for 7 min at 1,100 rpm onto a glass slides and were fixed and permeabilized as described above. The fixed cells were incubated with the following antibodies: mouse monoclonal antibody against HA (clone 12CA5) or Vpr (a gift from Dr Y. Ishizaka. The antibody was shown to recognize both Vpr WT and Q65R [12]) and a rabbit polyclonal antibody against VPRBP (Accurate Chemical and Scientific Corporation). The Duolink system provides oligonucleotide-labeled secondary antibodies (PLA probes) to each of the primary antibodies that, in combination with a DNA amplification-based reporter system, generate a signal only when the two primary antibodies are in close proximity. The signal from each detected pair of primary antibodies was visualized as a spot (please see the manufacturer's instructions for more details). Nuclei were delineated using Hoechst 33342.

### Cell cycle analysis

Cell cycle analysis was performed using propidium iodide staining and flow cytometry as previously described [12], [19].

### Immunoprecipitation and western blot

Immunoprecipitations using anti-HA-conjugated agarose beads were performed as previously described [26]. Analysis of proteins by western blot was performed as previously described [26].

### Bioluminescence resonance energy transfer (BRET) assays

HEK293T cells were transfected in 24-well plates with 10ng of the BRET donor plasmids pCDNA3.1_Rluc-MCS(MB), pCDNA3.1-Rluc-Vpr (WT), pCDNA3.1-Rluc-Vpr (R80A) or pCDNA3.1-Rluc-Vpr (Q65R) and increasing concentration (0 to 500 ng) of the BRET acceptor plasmids pCDNA3.1_eYFP-MCS(MB) or pCDNA3.1-eYFP-Vpr (WT) using Lipofectamine 2000. Two days after transfection, cells were harvested, washed twice in PBS, and aliquoted in two wells of a 96-well plate (Costar 3917). Total eYFP fluorescence was measured with an excitation wavelength of 485 nm and an emission wavelength at 520±10 nm. BRET was initiated by adding 5µM of the *renilla* luciferase substrate coelenterazine H (Prolume Ltd., Lakeside, AZ, USA). Luminescence was then measured 10 minutes later at 475±15 nm and BRET fluorescence was measured at 535±15 nm. All measurements were performed on a PheraStar microplate reader (BMG Labtech, Cary, NC, USA). BRET ratios were calculated using this formula: (emission at 535 nm/emission at 475 nm)-(background emission at 535nm/background emission at 475 nm), as previously described [60].

### Chromatin binding assays

Cells were lysed in triton lysis buffer (50 mM Tris pH 7.5, 150 mM NaCl, 0.5% Triton X-100, and complete protease inhibitors cocktail (Roche) for 15 minutes. Insoluble cell debris, including chromatin, was pelleted by centrifugation (2500g for 10 minutes). The supernatant was harvested and represented the soluble input control. Pellets were washed once with nuclease buffer (50 mM Tris pH 8.0, 5 mM CaCl_2_, and 100 µg/ml BSA), split in two, and resuspended in nuclease buffer alone or nuclease buffer containing 200 U/ml microccocal nuclease (New England Biolabs, Ipswich, MA, USA). Pellets were incubated for 30 minutes on ice and then centrifuged at 12000g for 10 minutes. The supernatant was harvested and represented the chromatin-bound fraction. The corresponding supernatant obtained in absence of nuclease was used to control for non-specific release. For immunoprecipitation experiments, soluble and nuclease-treated fractions were incubated with 25 µl of anti-HA-conjugated agarose beads (Sigma-Aldrich) for 2h at 4C. In some experiments, immunoprecipitations were supplemented with 25 µg/ml ethidium bromide to displace proteins from DNA [47].

## Supporting Information

Figure S1 Native Vpr and virally-encoded Vpr form nuclear foci. **A**) HeLa cells were transfected with plasmids expressing native Vpr. Two days after transfection, cells were fixed, permeabilized, and stained with monoclonal antibodies against Vpr (clone 8D1) and analyzed by confocal microscopy. **B**) HeLa cells were infected with VSV-G-pseudotyped viruses defective for Vpr expression (HxBru Vpr-) or expressing HA-tagged Vpr (HxBru HA-Vpr) at 100 cpm/cell. Two days after infection, cells were fixed, permeabilized, and stained with antibodies against HA (red), nucleoporin (blue) and VPRBP (green). Images were acquired by confocal microscopy. Images shown are representative of multiple fields that encompass minor and major phenotypes. C) Hela cells were infected at a MOI of 1.0 with VSV-G-pseudotyped viruses expressing Vpr WT (WT) or Vpr L23F (L23F) or expressing Vpr WT while harboring the L44P,F45S mutations in the p6 domain of Gag (LF/PS). Two days after infection, cells were fixed, permeabilized, and stained with monoclonal antibodies against Vpr (red) and nucleoporin (blue). Images were acquired by confocal microscopy. Images shown are representative of multiple fields.
(2.10 MB PDF)

Figure S2 Vpr Q65R is not in close proximity to VPRBP. HeLa cells were transfected with plasmids expressing GFP alone (WPI) or co-expressing GFP and Vpr WT (WPI-Vpr WT) or GFP and Vpr Q65R (WPI-Vpr Q65R). In situ proximity ligation assay (PLA) was performed on HeLa cells stained with a mouse monoclonal antibody against Vpr and a rabbit polyclonal antibody against VPRBP. A flurochrome-labeled probe (red) was then used to reveal locations of close proximity between the two proteins in GFP-expressing cells (green). Hoechst 33342 was used to highlight nuclei (cyan). Images were acquired by confocal microscopy with a 63× objective. Images shown are representative of multiple fields.
(1.58 MB PDF)

Figure S3 Vpr nuclear foci do not co-localize with SC35 or PML. HeLa cells were transduced with lentiviral vectors expressing HA-Vpr. Two days after transduction, cells were fixed, permeabilized, and stained with **A**) antibodies against HA (red) and SC35 (green) or **B**) antibodies against HA (red) and PML (green). Images were acquired by confocal microscopy. Images shown are representative of multiple fields.
(0.63 MB PDF)

Figure S4 Depletion of VPRBP inhibits formation of DNA repair foci but not of Vpr nuclear foci. **A**) HeLa cells were transfected with control scrambled siRNA or siRNA targeting VPRBP. Twenty-four hours after transfection, cells were transduced with a lentiviral vector expressing HA-Vpr. One day after transduction, cells were fixed, permeabilized, and stained with antibodies against HA (red), γ-H2AX (green) and 53BP1 (blue). DAPI was used to highlight nuclei (cyan). Images were acquired by confocal microscopy. Images shown are representative of multiple fields. Yellow arrows highlight examples of punctuate co-localization. **B**) The numbers of γ-H2AX or 53BP1 foci per cell in A) were quantified and cells with greater than 10 foci were considered positive. Results depicted in the graph are the means of three independent experiments. Error bars represent standard deviations.
(1.28 MB PDF)

Figure S5 Analysis of the effect of blocking the Vpr-p6 interaction on Vpr nuclear foci formation and induction of G2 arrest. **A**) HeLa cells were transfected with a plasmid expressing Vpr L23F. Two days after transfection, cells were fixed, permeabilized, and stained with monoclonal antibodies against Vpr (clone 8D1) and nucleoporin (blue) and analyzed by confocal microscopy. **B**) HeLa cells were co-transfected with the packaging plasmid psPAX2 encoding Gag-Pol, Tat, and Rev and with plasmids expressing Vpr WT or Vpr L23F. Two days after transfection, cells were fixed, permeabilized, and stained with antibodies against Vpr (red), nucleoporin (blue) and p24 (green). Images were acquired by confocal microscopy. Images shown are representative of multiple fields. **C**) HEK293T cells were cotransfected with plasmids expressing GFP, Vpr (WT or L23F) and Gag-Pol or with an empty plasmid control as indicated. Forty-eight hours after transfection, cell cycle analysis was performed by flow cytometry using propidium iodide staining. Percentages of G1 and G2/M cell populations were determined using the ModFit software. **D**) Hela cells were infected at a multiplicity of infection of 1.0 with VSV-G-pseudotyped viruses defective for Vpr expression (HxBru Vpr-) or expressing Vpr WT in the context of wild type p6 (HxBru VprWT) or mutated p6 (HxBru VprWT LF/PS). Mock-infected cells were used as a negative control. Forty-eight hours after infection, cell cycle analysis of HIV-1-expressing cells was performed by flow cytometry using FITC-conjugated anti-p24 monoclonal antibodies and propidium iodide staining. Percentages of p24+ cells in G1 and G2/M were determined using the ModFit software.
(2.70 MB PDF)

Figure S6 Vpr nuclear foci are long-lived and display limited exchange of Vpr molecules. **A**) HeLa cells were transfected with a plasmid expressing eYFP-Vpr WT. Two days after transfection, the location of eYFP-Vpr was monitored by time-lapse spinning-disk confocal microscopy in living cells. Images were acquired with a 60× objective at intervals of 5 seconds for 15 minutes. One hundred and ten Z cross-sections were taken for each time point. Vpr foci were tracked using the Volocity software v.5.2.1. Movement tracks of some foci are depicted in color on the orthogonal sections of the images acquired at time 0. The graph on the right panel shows mean fluorescence intensity for each tracked focus over time. **B**) Hela cells transfected as in A) were subjected to FRAP (fluorescence recovery after photobleaching) assays. Regions of interest included photobleached background (green), photobleached eYFP-Vpr focus (red) and control eYFP-Vpr focus (blue). Images were acquired by spinning-disk confocal microscopy at 5 seconds intervals for 400 seconds. After 50 seconds, the indicated regions of interest were partially photobleached to allow detection and tracking of mobile eYFP-Vpr foci. The graph on the right panel shows mean fluorescence intensity for each region of interest over time. Results shown are representative of multiple experiments.
(1.06 MB PDF)

Figure S7 Localization and G2 arrest activity of the Vpr mutants V57L and R62P. **A**) HeLa cells were transfected with plasmids expressing HA-tagged Vpr (V57L) and Vpr (R62P). Forty-eight hours after transfection, cells were fixed, permeabilized, and stained with antibodies against HA (red) and nucleoporin (blue). Images were acquired by confocal microscopy. Images shown are representative of multiple fields. 60% of cells expressing HA-Vpr (V57L) could form nuclear foci while the remaining 40% displayed perinuclear accumulation with reduced or absence of nucleoporin staining. 20% of cells expressing HA-Vpr (R62P) displayed an exclusive nuclear localization while the remaining 80% of cells showed accumulation of Vpr in the cytoplasm. In all cases, HA-Vpr (R62P) did not form nuclear foci. **B**) HEK293T cells were co-transfected with a plasmid expressing GFP and a plasmid expressing HA-Vpr (WT), HA-Vpr (V57L), or HA-Vpr (R62P). An empty plasmid was used as negative control (mock). Forty-eight hours after transfection, cell cycle analysis was performed by flow cytometry using propidium iodide staining. Percentages of G1 and G2/M cell populations were determined using the ModFit software.
(2.14 MB PDF)

Video S1 Vpr nuclear foci are mobile nuclear bodies. HeLa cells were transfected with a plasmid expressing eYFP-Vpr WT. Two days after transfection, the location of eYFP-Vpr was monitored by time-lapse confocal microscopy in living cells. Images were acquired with a 63× objective at intervals of 2 seconds for two minutes. The frame rate was accelerated 10 times to facilitate visualization. Note that the presence of eYFP-Vpr at the nuclear membrane allows delineation of the nucleus.
(8.04 MB AVI)

Video S2 Vpr nuclear foci are mobile nuclear bodies. HeLa cells were transfected with a plasmid expressing eYFP-Vpr WT. Two days after transfection, the location of eYFP-Vpr was monitored by time-lapse confocal microscopy in living cells. Images were acquired with a 63× objective at intervals of 2 seconds for two minutes. The frame rate was accelerated 10 times to facilitate visualization. Note that the presence of eYFP-Vpr at the nuclear membrane allows delineation of the nucleus.
(0.96 MB AVI)
