# Supplementary figures and images for: Formation of Mobile Chromatin-Associated Nuclear Foci Containing HIV-1 Vpr and VPRBP Is Critical for the Induction of G2 Cell Cycle Arrest
Source: PLoS Pathog. 2010 Sep 2;6(9):e1001080. doi: 10.1371/journal.ppat.1001080 (PMC2932712; doi:10.1371/journal.ppat.1001080)

**A**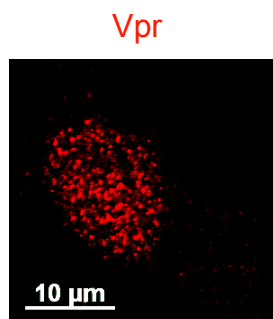**C**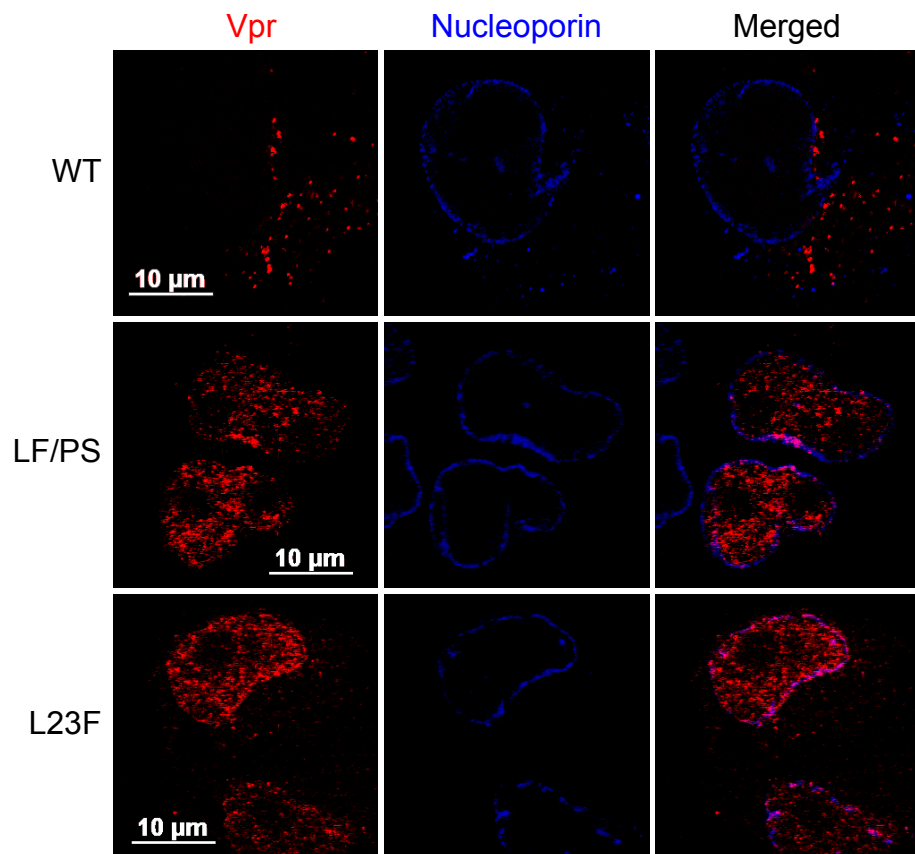**B**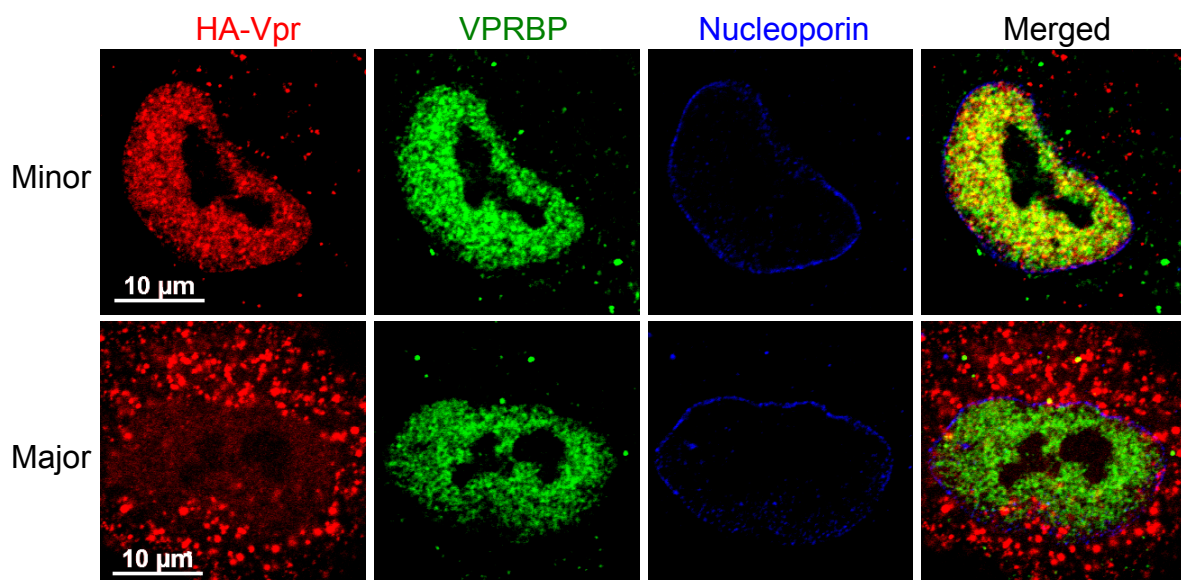

Supplement: Figure S1 — Native Vpr and virally-encoded Vpr form nuclear foci. A) HeLa cells were transfected with plasmids expressing native Vpr. Two days after transfection, cells were fixed, permeabilized, and stained with monoclonal antibodies against Vpr (clone 8D1) and analyzed by confocal microscopy. B) HeLa cells were infected with VSV-G-pseudotyped viruses defective for Vpr expression (HxBru Vpr-) or expressing HA-tagged Vpr (HxBru HA-Vpr) at 100 cpm/cell. Two days after infection, cells were fixed, permeabilized, and stained with antibodies against HA (red), nucleoporin (blue) and VPRBP (green). Images were acquired by confocal microscopy. Images shown are representative of multiple fields that encompass minor and major phenotypes. C) Hela cells were infected at a MOI of 1.0 with VSV-G-pseudotyped viruses expressing Vpr WT (WT) or Vpr L23F (L23F) or expressing Vpr WT while harboring the L44P,F45S mutations in the p6 domain of Gag (LF/PS). Two days after infection, cells were fixed, permeabilized, and stained with monoclonal antibodies against Vpr (red) and nucleoporin (blue). Images were acquired by confocal microscopy. Images shown are representative of multiple fields. (2.10 MB PDF) [file ppat.1001080.s001.pdf]

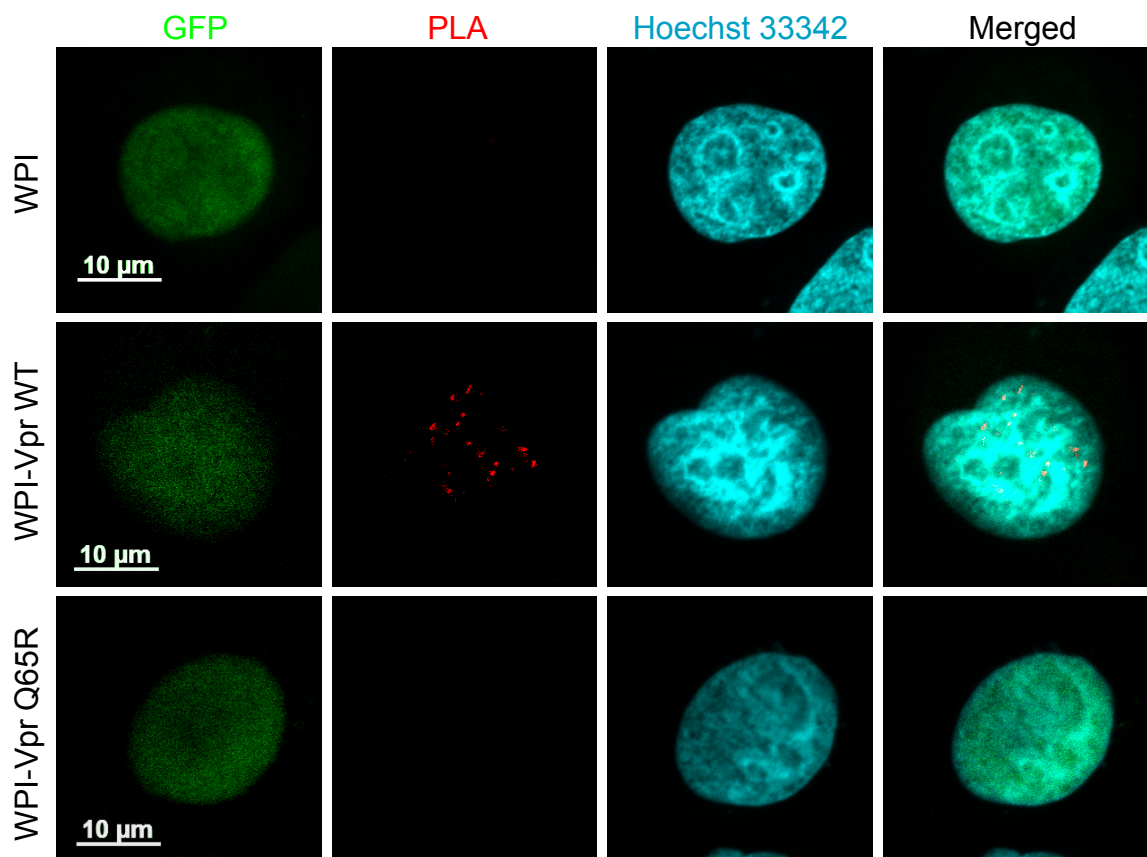

Supplement: Figure S2 — Vpr Q65R is not in close proximity to VPRBP. HeLa cells were transfected with plasmids expressing GFP alone (WPI) or co-expressing GFP and Vpr WT (WPI-Vpr WT) or GFP and Vpr Q65R (WPI-Vpr Q65R). In situ proximity ligation assay (PLA) was performed on HeLa cells stained with a mouse monoclonal antibody against Vpr and a rabbit polyclonal antibody against VPRBP. A flurochrome-labeled probe (red) was then used to reveal locations of close proximity between the two proteins in GFP-expressing cells (green). Hoechst 33342 was used to highlight nuclei (cyan). Images were acquired by confocal microscopy with a 63× objective. Images shown are representative of multiple fields. (1.58 MB PDF) [file ppat.1001080.s002.pdf]

**A**

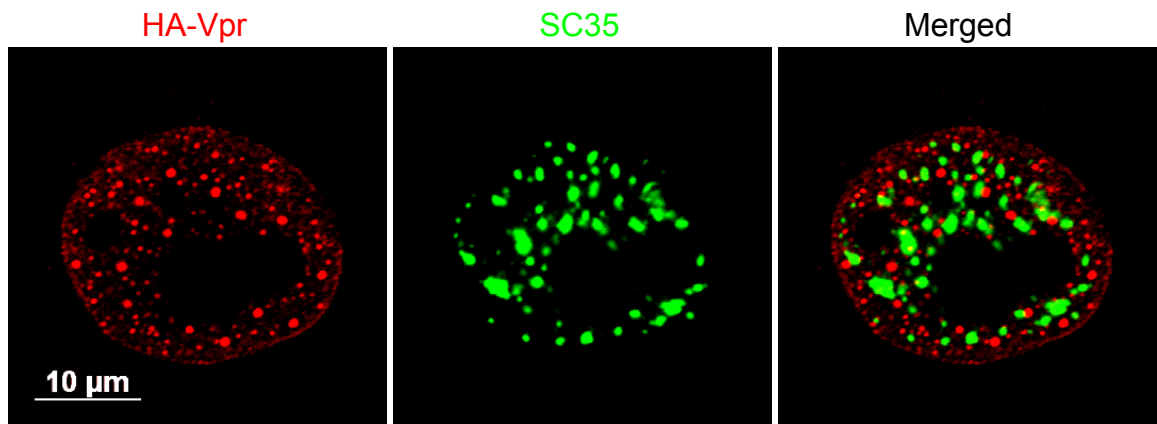

**B**

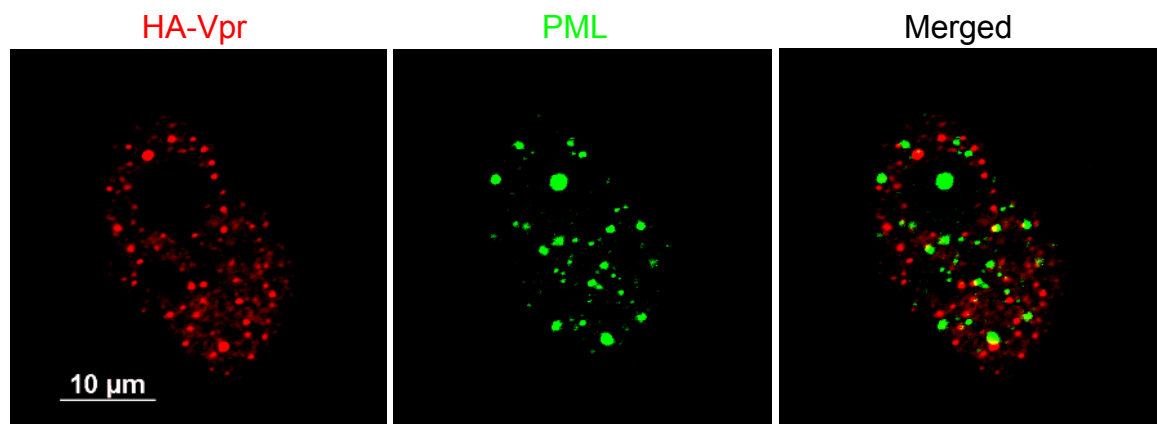

Supplement: Figure S3 — Vpr nuclear foci do not co-localize with SC35 or PML. HeLa cells were transduced with lentiviral vectors expressing HA-Vpr. Two days after transduction, cells were fixed, permeabilized, and stained with A) antibodies against HA (red) and SC35 (green) or B) antibodies against HA (red) and PML (green). Images were acquired by confocal microscopy. Images shown are representative of multiple fields. (0.63 MB PDF) [file ppat.1001080.s003.pdf]

**A**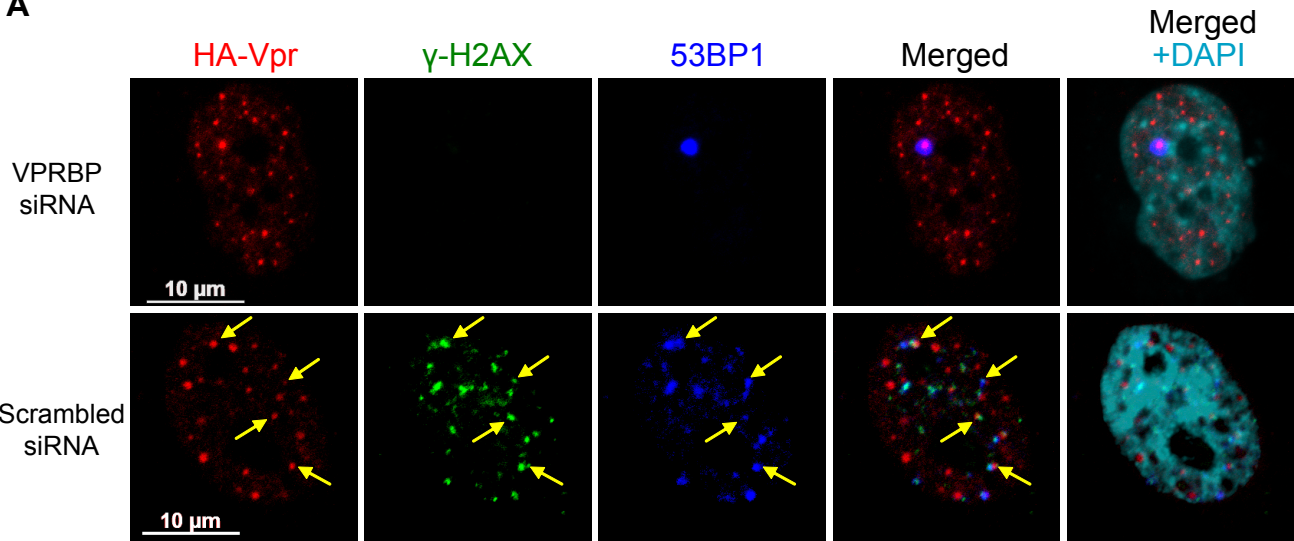**B**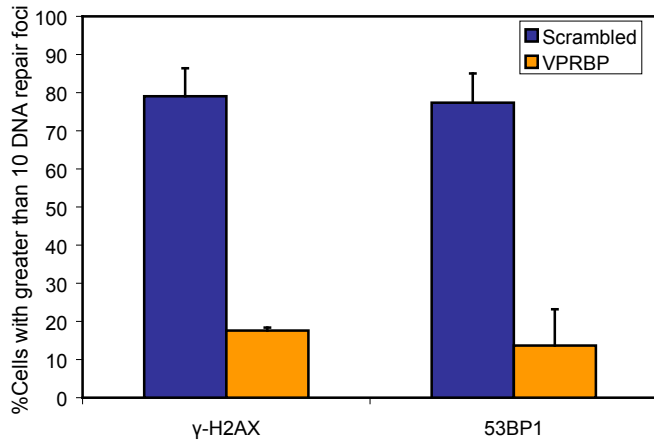

Supplement: Figure S4 — Depletion of VPRBP inhibits formation of DNA repair foci but not of Vpr nuclear foci. A) HeLa cells were transfected with control scrambled siRNA or siRNA targeting VPRBP. Twenty-four hours after transfection, cells were transduced with a lentiviral vector expressing HA-Vpr. One day after transduction, cells were fixed, permeabilized, and stained with antibodies against HA (red), γ-H2AX (green) and 53BP1 (blue). DAPI was used to highlight nuclei (cyan). Images were acquired by confocal microscopy. Images shown are representative of multiple fields. Yellow arrows highlight examples of punctuate co-localization. B) The numbers of γ-H2AX or 53BP1 foci per cell in A) were quantified and cells with greater than 10 foci were considered positive. Results depicted in the graph are the means of three independent experiments. Error bars represent standard deviations. (1.28 MB PDF) [file ppat.1001080.s004.pdf]

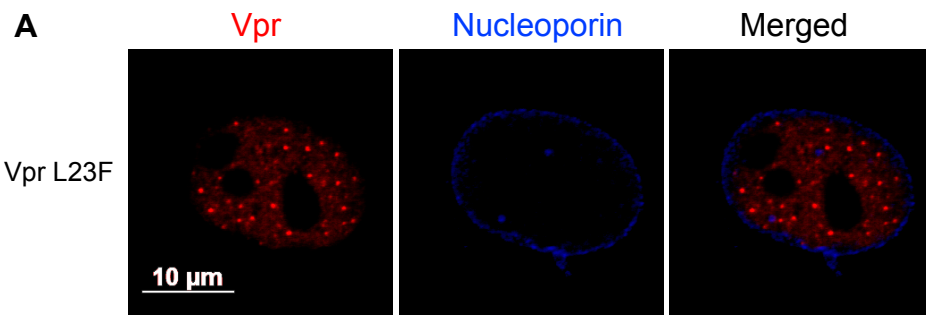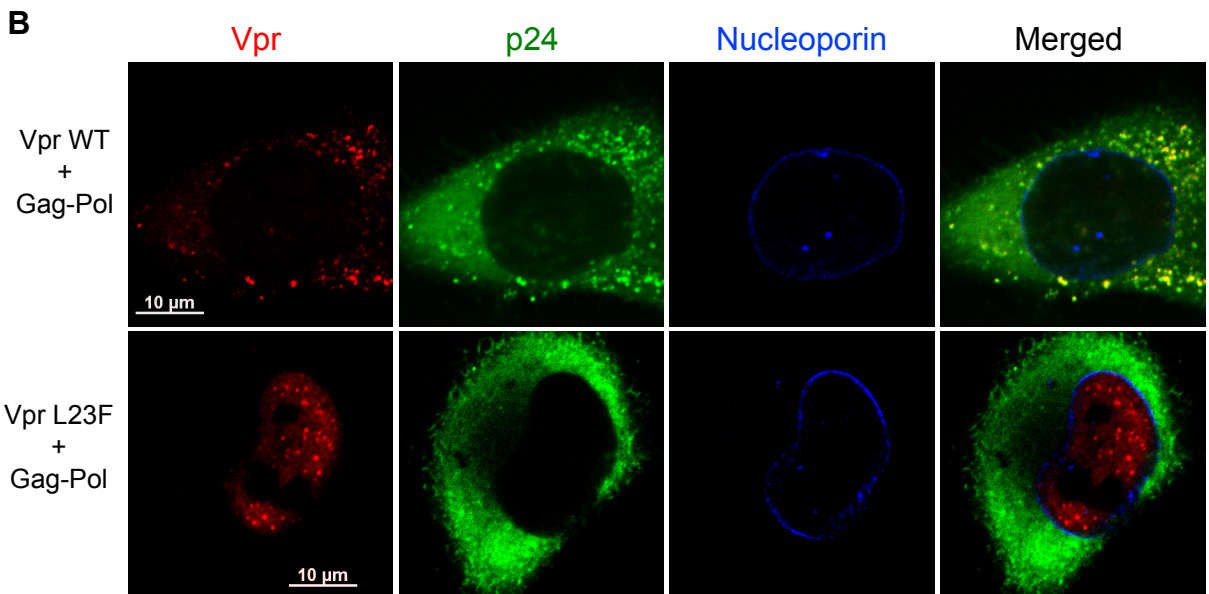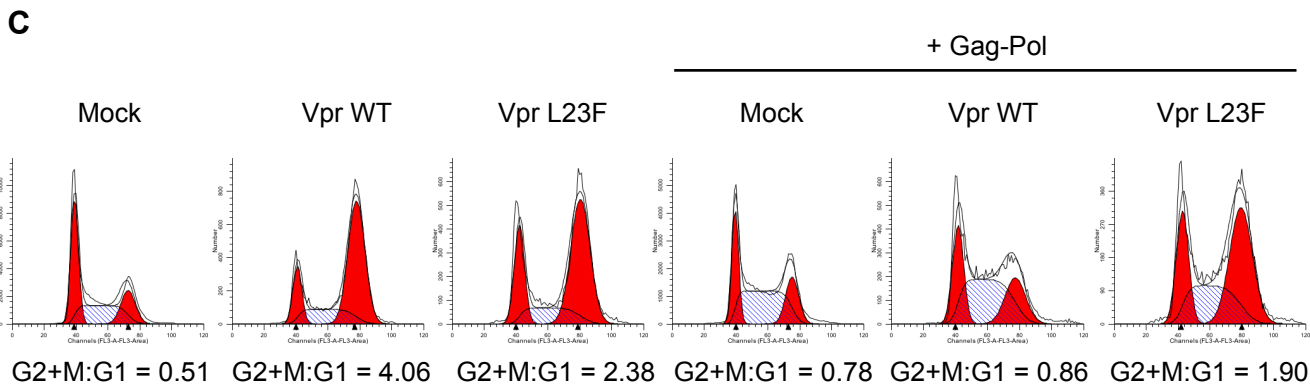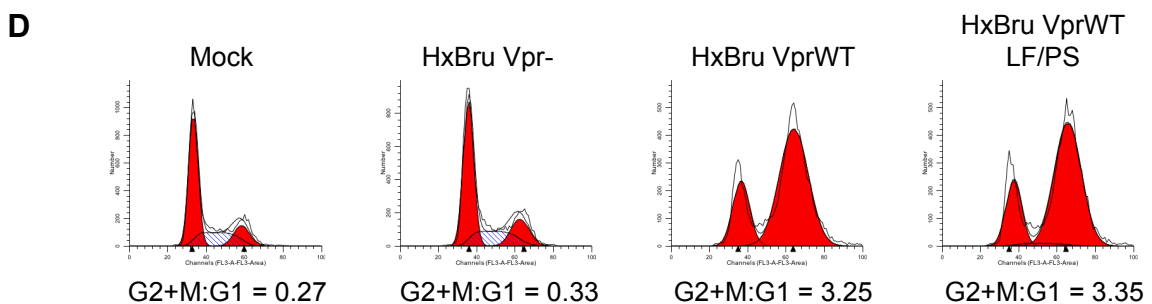

Supplement: Figure S5 — Analysis of the effect of blocking the Vpr-p6 interaction on Vpr nuclear foci formation and induction of G2 arrest. A) HeLa cells were transfected with a plasmid expressing Vpr L23F. Two days after transfection, cells were fixed, permeabilized, and stained with monoclonal antibodies against Vpr (clone 8D1) and nucleoporin (blue) and analyzed by confocal microscopy. B) HeLa cells were co-transfected with the packaging plasmid psPAX2 encoding Gag-Pol, Tat, and Rev and with plasmids expressing Vpr WT or Vpr L23F. Two days after transfection, cells were fixed, permeabilized, and stained with antibodies against Vpr (red), nucleoporin (blue) and p24 (green). Images were acquired by confocal microscopy. Images shown are representative of multiple fields. C) HEK293T cells were cotransfected with plasmids expressing GFP, Vpr (WT or L23F) and Gag-Pol or with an empty plasmid control as indicated. Forty-eight hours after transfection, cell cycle analysis was performed by flow cytometry using propidium iodide staining. Percentages of G1 and G2/M cell populations were determined using the ModFit software. D) Hela cells were infected at a multiplicity of infection of 1.0 with VSV-G-pseudotyped viruses defective for Vpr expression (HxBru Vpr-) or expressing Vpr WT in the context of wild type p6 (HxBru VprWT) or mutated p6 (HxBru VprWT LF/PS). Mock-infected cells were used as a negative control. Forty-eight hours after infection, cell cycle analysis of HIV-1-expressing cells was performed by flow cytometry using FITC-conjugated anti-p24 monoclonal antibodies and propidium iodide staining. Percentages of p24+ cells in G1 and G2/M were determined using the ModFit software. (2.70 MB PDF) [file ppat.1001080.s005.pdf]

**A**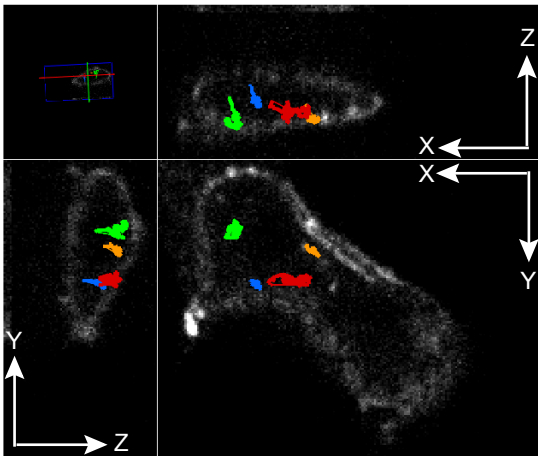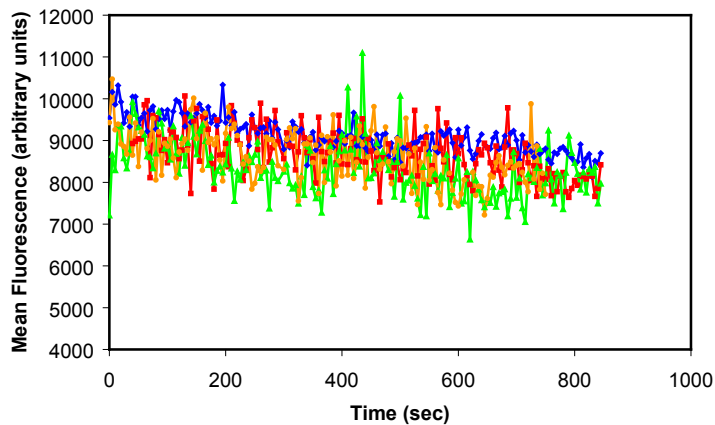**B**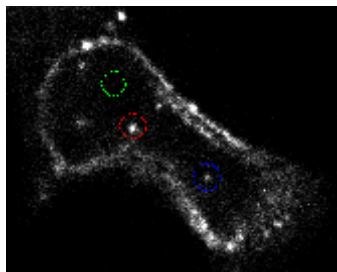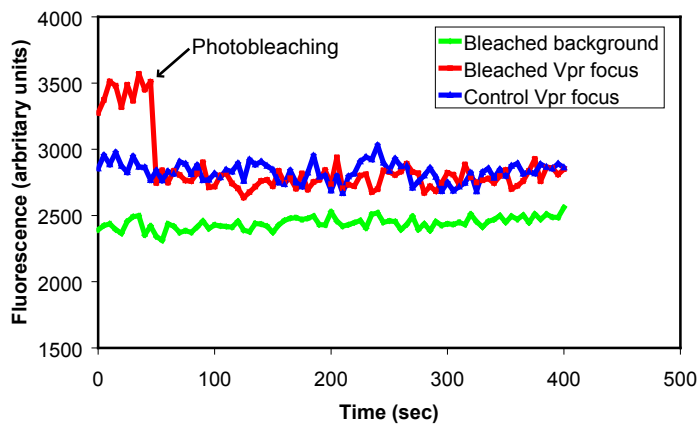

Supplement: Figure S6 — Vpr nuclear foci are long-lived and display limited exchange of Vpr molecules. A) HeLa cells were transfected with a plasmid expressing eYFP-Vpr WT. Two days after transfection, the location of eYFP-Vpr was monitored by time-lapse spinning-disk confocal microscopy in living cells. Images were acquired with a 60× objective at intervals of 5 seconds for 15 minutes. One hundred and ten Z cross-sections were taken for each time point. Vpr foci were tracked using the Volocity software v.5.2.1. Movement tracks of some foci are depicted in color on the orthogonal sections of the images acquired at time 0. The graph on the right panel shows mean fluorescence intensity for each tracked focus over time. B) Hela cells transfected as in A) were subjected to FRAP (fluorescence recovery after photobleaching) assays. Regions of interest included photobleached background (green), photobleached eYFP-Vpr focus (red) and control eYFP-Vpr focus (blue). Images were acquired by spinning-disk confocal microscopy at 5 seconds intervals for 400 seconds. After 50 seconds, the indicated regions of interest were partially photobleached to allow detection and tracking of mobile eYFP-Vpr foci. The graph on the right panel shows mean fluorescence intensity for each region of interest over time. Results shown are representative of multiple experiments. (1.06 MB PDF) [file ppat.1001080.s006.pdf]

**A**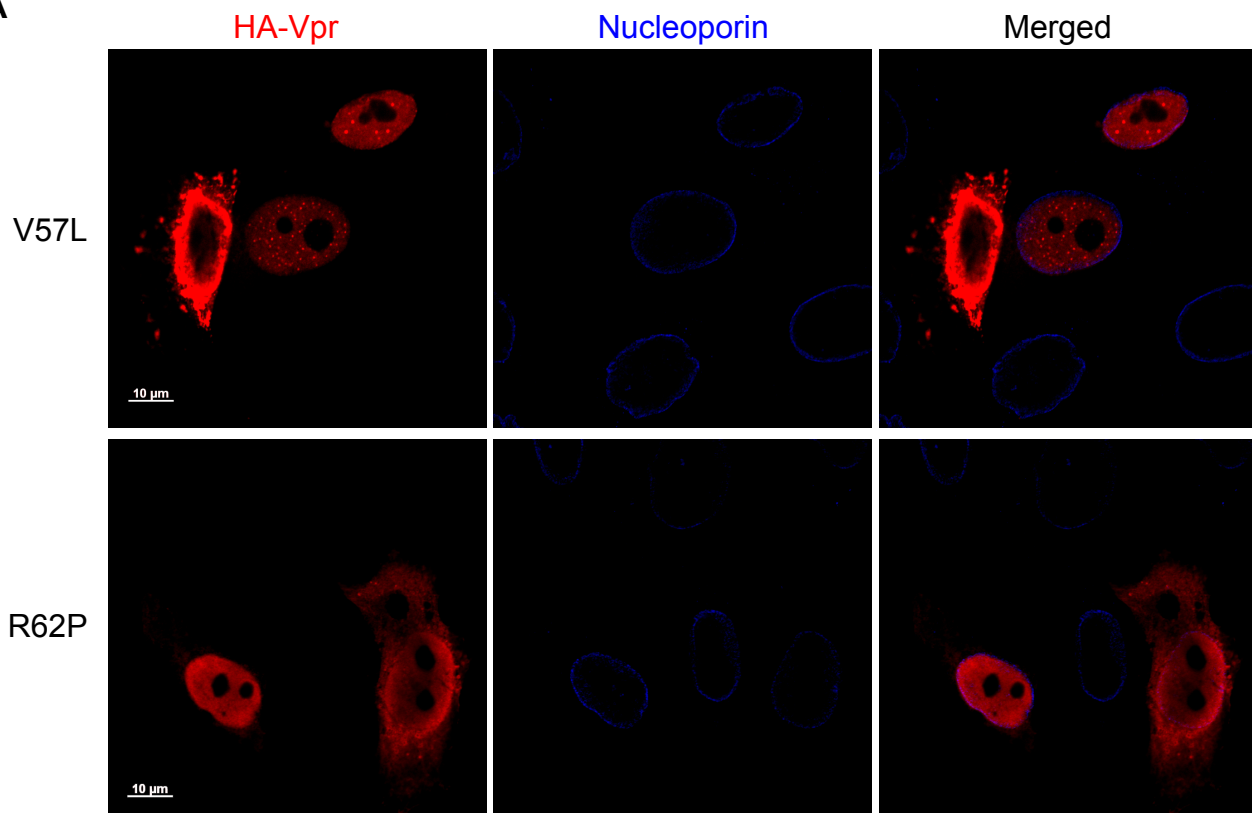**B**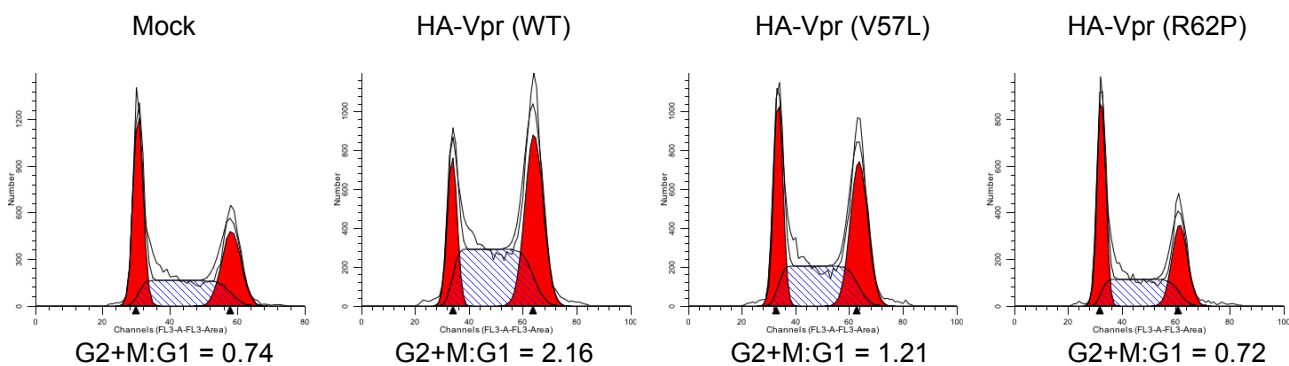

Supplement: Figure S7 — Localization and G2 arrest activity of the Vpr mutants V57L and R62P. A) HeLa cells were transfected with plasmids expressing HA-tagged Vpr (V57L) and Vpr (R62P). Forty-eight hours after transfection, cells were fixed, permeabilized, and stained with antibodies against HA (red) and nucleoporin (blue). Images were acquired by confocal microscopy. Images shown are representative of multiple fields. 60% of cells expressing HA-Vpr (V57L) could form nuclear foci while the remaining 40% displayed perinuclear accumulation with reduced or absence of nucleoporin staining. 20% of cells expressing HA-Vpr (R62P) displayed an exclusive nuclear localization while the remaining 80% of cells showed accumulation of Vpr in the cytoplasm. In all cases, HA-Vpr (R62P) did not form nuclear foci. B) HEK293T cells were co-transfected with a plasmid expressing GFP and a plasmid expressing HA-Vpr (WT), HA-Vpr (V57L), or HA-Vpr (R62P). An empty plasmid was used as negative control (mock). Forty-eight hours after transfection, cell cycle analysis was performed by flow cytometry using propidium iodide staining. Percentages of G1 and G2/M cell populations were determined using the ModFit software. (2.14 MB PDF) [file ppat.1001080.s007.pdf]
